# Zero-Degree Celsius Capillary Electrophoresis Electrospray Ionization for Hydrogen Exchange Mass Spectrometry

**DOI:** 10.1021/acs.analchem.2c03893

**Published:** 2022-12-22

**Authors:** Jordan T. Aerts, Per E. Andrén, Erik T. Jansson

**Affiliations:** † Department of Pharmaceutical Biosciences, Uppsala University, Uppsala751 24, Sweden; ‡ Science for Life Laboratory, Spatial Mass Spectrometry, Uppsala University, Uppsala751 24, Sweden

## Abstract

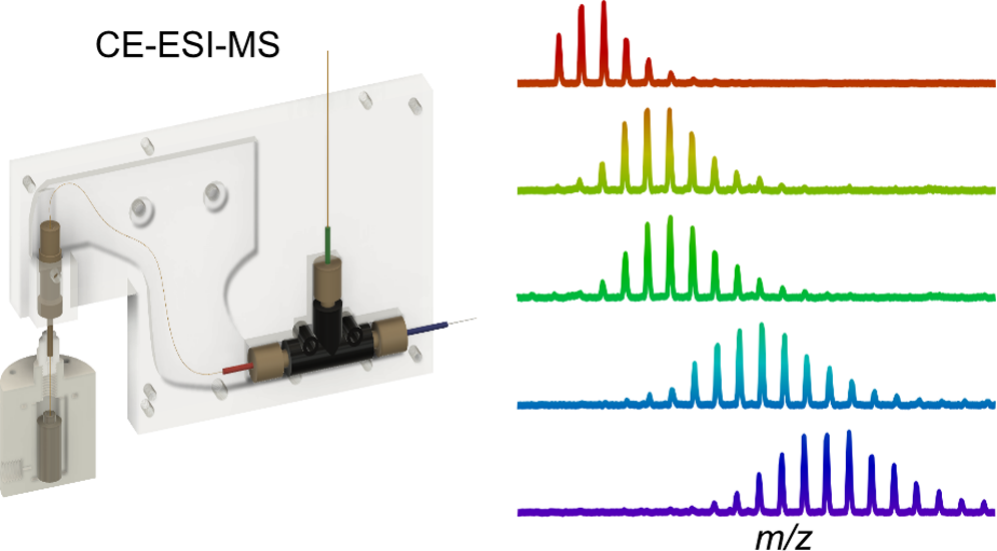

Currently, fast liquid chromatographic separations at low temperatures are exclusively used for the separation of peptides generated in hydrogen deuterium exchange (HDX) workflows. However, it has been suggested that capillary electrophoresis may be a better option for use with HDX. We performed in solution HDX on peptides and bovine hemoglobin (Hb) followed by quenching, pepsin digestion, and cold capillary electrophoretic separation coupled with mass spectrometry (MS) detection for benchmarking a laboratory-built HDX–MS platform. We found that capillaries with a neutral coating to eliminate electroosmotic flow and adsorptive processes provided fast separations with upper limit peak capacities surpassing 170. In contrast, uncoated capillaries achieved 30% higher deuterium retention for an angiotensin II peptide standard owing to faster separations but with only half the peak capacity of coated capillaries. Data obtained using two different separation conditions on peptic digests of Hb showed strong agreement of the relative deuterium uptake between methods. Processed data for denatured versus native Hb after deuterium labeling for the longest timepoint in this study (50,000 s) also showed agreement with subunit interaction sites determined by crystallographic methods. All proteomic data are available under DOI: 10.6019/PXD034245.

Hydrogen exchange studies of proteins, have since its inception in the 1950s, have become a key tool for structural biologists.^1,2^ Hydrogen deuterium exchange (HDX) is a well-established technique often combined with mass spectrometry (MS) for studying protein dynamics and interactions in solution.^3^ When placed into a solution of deuterium oxide, amide hydrogens of the peptide backbone of a protein will exchange with deuterons (in exchange) at a rate primarily determined by the presence and stability of local hydrogen bonding.^4^ The exchange is quenched by reducing the temperature to 0 °C and the pH to 2.5. This combined reduction in temperature and pH results in a decrease of the exchange rate by more than 5 orders of magnitude, and subsequent manipulation of the samples is typically performed under quench conditions to minimize the loss of the deuterium label (out exchange) from the peptide amide backbone prior to the measurement of deuterium incorporation.^4^ Sample handling steps following the quenching step have grown with the complexity of the system being investigated and primarily include digestion by acid-resistant proteases followed by desalting/concentration with a trap column but can also involve additional steps to facilitate the protein digestion, such as protein denaturation, chemical or electrochemical reduction of disulfide bonds, detergent depletion and lipid stripping from membrane proteins, and deglycosylation of glycosylated proteins.^5−13^ Today, HDX–MS workflows are dominated by variants of liquid chromatography (LC)–MS approaches based on a Peltier-cooled ultra-performance liquid chromatography (UPLC) module.^14^ Costly commercial platforms for HDX–MS are abundant in structural biology research, where they offer automated, online sample handling and analysis by low-temperature UPLC separations. The main drawbacks of these systems are their cost, and due to the low temperatures, high backpressures (up to ∼20,000 psi) because of increased mobile phase viscosity, and reduced separation efficiency of LC (a result of resistance of mass transfer).^15,16^ This resistance to mass transfer not only degrades the separation efficiency of LC but also results in an increased risk of carryover of injected material from prior injections as the interaction between peptides and protease, trap, and analytical column stationary phase becomes more difficult to disrupt. The carryover of material is detrimental to HDX experiments as retained peptides lose deuterons as they are exposed to 100% H_2_O solvents, only to then elute during subsequent separations, confounding the apparent uptake kinetics.^17,18^ Despite all these drawbacks, LC remains the workhorse of HDX–MS workflows although there have been a handful of attempts to employ capillary electrophoresis (CE) in HDX experiments.

CE is a well-established method for the separation of numerous classes of molecules and is a valuable method for proteomics research.^19−21^ CE separates based on the differential mobility of analytes in an electric field with a velocity dependent on their size and charge (eq 1).^22^ In the beginning of the 1990s, CE–MS was used for tryptic digests of proteins and protein complexes. Since then, CE–MS has demonstrated 10–100 times better sensitivity than reversed phase LC–MS for the measurement of peptides and proteoforms^19,23−25^ Recently, researchers in the field of HDX–MS have been alluding to the potential advantages of using CE for HDX–MS workflows.^26−29^ There have been several studies where CE–MS has been used for HDX experiments but these studies employed deuterated background electrolyte (BGE) or deuterated sheath-flow additives and, as such, offer limited application to structural investigations.^30−32^ Microchip electrophoresis has been utilized for the separation of several proteins following label, quench, and digestion steps, and though the separation was performed at ambient temperature, the speed of the separations allowed for similar deuterium retention to cold LC separations.^16^ One advantage of CE that could make it suitable for HDX is that, in contrast to LC, the separation performance is, theoretically, improved at lower temperatures, for two main reasons:

First, low temperatures result in increased viscosity (η) of the BGE,^33^ reducing any longitudinal diffusion of the analytes, as shown in the equation for electrophoretic mobility μ given by the Einstein relation
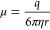
1where *q* is the effective charge and *r* is the hydrodynamic radius of the analyte. One trade off of this advantage is the requirement for higher field strengths to maintain the same CE current as achieved in separations at room temperature or even warmer conditions.

Second, in CE, the number of theoretical plates *N* can be defined as
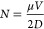
2where *V* is the potential difference driving the separation, μ is the electrophoretic mobility of the analyte, and *D* is the diffusivity of the analyte. In turn, the diffusivity of an analyte in a liquid with a low Reynolds number can be determined from the Stokes–Einstein equation
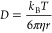
3where *T* is the temperature and *k*_B_ is Boltzmann’s constant. As shown by Ma and Horváth,^33^ combining eqs 1–3 gives
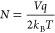
4

Hence, the plate number *N* is directly proportional to the voltage applied in CE and inversely proportional to the temperature *T*. It should be noted that a reduction of temperature from ambient to 0 °C results in merely ∼10% increase of *N*. While the theoretical improvement is small, CE appears to suffer less from the low temperatures used to reduce out exchange in HDX experiments, in comparison to LC, where the backing pressure during cold separation may approach the limits of what the system fittings can sustain.

In this seminal study, we present proof of principal of in-solution deuterium labeling of peptides and proteins followed by quenching, digestion, and separation in fused-silica capillaries at subzero-degrees Celsius temperatures using CE–MS. The platform is cost-efficient and easy to implement with any MS.

## Materials and Methods

### Chemicals

Sodium hydroxide (∼3 mm flakes, 97% Aldrich 48024), high-performance liquid chromatography (HPLC) peptide standard mix (Sigma H2016-1VL), hemoglobin (Hb) from bovine erythrocytes (EMD Millipore 374834-10 g), ammonium persulfate (APS) (≥98% Sigma 248614-5 g), and bradykinin (BK) acetate salt (≥98% Sigma B3259-5 mg) were obtained from Sigma-Aldrich (Darmstadt, Germany). Ammonium bicarbonate (99% Acros, 393210010), acetonitrile (ACN) (Optima, Fisher A955-212), H_2_O (Optima, Fisher W6-4), formic acid (99+% Pierce 28950), formic acid (Optima, Fisher A117-50), acetic acid (Fisher A113-10x1AMP), dimethylformamide (≥99.5%, Fisher D/3846/17), ammonium acetate (Optima, Fisher A114-50), D_2_O (99.8%, Thermo Cat 166300100), urea (99.5%, Acros, 140750010), pepsin from porcine gastric mucosa (Acros, 417071000), and methanol (Optima, Fisher A456-4) were obtained from Fisher Scientific (Göteborg, Sweden).

### Capillary Electrophoresis

CE separations were performed, and gas phase ions generated using a coaxial sheath flow CE–ESI interface previously described,^34^ with the modifications provided below.

A direct current high voltage power supply (HVPS; HPS100-40-0.4, Beijing Excellent Innovate HD Electronics Company, Beijing, China) was manually operated to ramp the voltage over ∼7 s to 20 kV unless otherwise specified. Stable current was observed during the separations, and an Ohm’s plot shows the separations occurred within the linear region of the voltage–current curve (Figure S1). Separations were performed in the anode to cathode mode using 24 cm long fused silica separation capillaries with 105 μm outer diameter (OD) and 40 μm inner diameter (ID) (TSP040105, Polymicro Technologies, Phoenix, AZ). Hydrodynamic sample injections were performed using 5 psi N_2_ (g) backing for 3 s for both capillaries used in this study (a pneumatic system diagram is shown in Figure S2). The electrical circuit was connected to earth ground via the stainless-steel needle of the sheath liquid syringe. For uncoated bare fused silica (BFS) separations, the separation capillary was conditioned between injections for 6 min with 3/1 (v/v) H_2_O/ACN, 10 min with 3/1 (v/v) 0.1 M NaOH/ACN, 10 min with 3/1 (v/v) H_2_O/ACN, and 10 min with BGE 3/1 (v/v) 1% formic acid (aq)/ACN delivered at 20 psi of N_2_ (g) by connecting the separation capillary inlet to a laboratory built acrylic holder for solution vials using 1/4-28 male to Luer lock assemblies (P-683, Idex Health & Science, Oak Harbor, WA; Figures S3–S5). For linear polyacrylamide (LPA)-coated capillaries, 10 min of BGE at 20 psi was used to condition the capillary between sample and standard injections. The capillary inlet was rinsed with water after NaOH flushing and sample injection (Figure S6).

The LPA capillaries were prepared, as previously described.^35^ Note that it is important to use fresh APS for the coating procedure. One suggestion is to prepare aliquots of 5% APS upon receipt of APS and store them at −20 °C for future use.

### Electrospray Ionization

**Figure 1:**
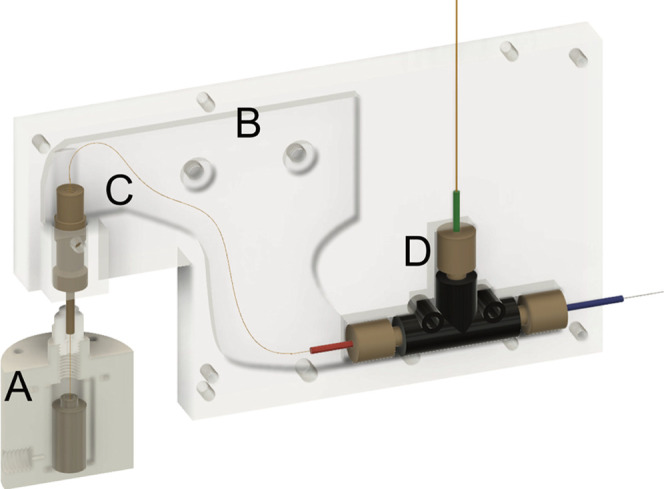
Layout of the Peltier-cooled CE housing attached to a sample pod for injection. (A) Gas-tight acrylic pod containing a small volume insert for hydrodynamic sample injection. (B) Half of the aluminum housing to which the Peltier element is attached. (C) Separation capillary inlet assembly. (D) Coaxial sheath flow nano-electrospray emitter.

A PEEK tee assembly (P-727, Idex Health & Science, Oak Harbor, WA) was housed in an aluminum block for thermal regulation and attached to a piece of optical breadboard fastened to a linear ball screw gantry stage (FSL40XYZ-L, Fuyu Technology, Chengdu, China; Figures 1 and S4). The stage was controlled by motion control software (AMC4030, Fuyu Technology, Chengdu, China) to position the ESI emitter at the mass spectrometer inlet. Stainless-steel hypodermic tubing (133 μm ID, 261 μm OD) of length 50 mm (Hamilton Company, Reno, Nevada) was used for the ESI emitter assembly. A 2 kV electrospray voltage was applied directly to the stainless-steel tubing and controlled using MassLynx software (Waters Corporation, Manchester, United Kingdom). The sheath liquid flow rate was 500 nL/min using a solution comprising 50% MeOH, 0.1% formic acid, and 50 nM leu-enk. The electrospray was visually monitored with a Dino-Lite long working distance microscope (AM4113TL, ANMO Electronics Corporation, New Taipei City, Taiwan).

### Thermal Regulation

A Peltier cooling module (APHC-12704-S, European Thermodynamics Limited, Leicestershire, United Kingdom) was mounted to the surface of an in-house designed (Autodesk Fusion, Autodesk, San Rafael, CA) and machined (DMU 70, DMG MORI, Nagoya, Japan) aluminum block holding the ESI emitter. A liquid CPU cooler (Hydro Series H150i Pro RGB 360 mm, Corsair, Fremont, CA) operated in extreme mode with Corsair iCUE software was fastened to a small aluminum heat spreader on the hot side of the Peltier cooling module to facilitate heat exchange (Figure S7, the CPU cooler is seen attached in panel B). The Peltier cooling module was controlled using a small OEM precision Peltier temperature controller (TEC-1091, Meerstetter Engineering, Rubigen, Switzerland) coupled with TEC Service software (Meerstetter Engineering, Rubigen, Switzerland).

### Mass Spectrometry

Generated ions were analyzed on a Synapt G2–Si (Waters Corporation, Manchester, United Kingdom) operated in positive ion mode with a mass range of 50–1200 Da in resolution mode using a desolvation temperature of 22 °C and 250 mL/min N_2_ (g) cone gas flow rate. The instrument was calibrated on the day of use using an appropriate dilution of sodium iodide (18600791-3, Waters Corporation, Manchester, United Kingdom) in 50% MeOH and 0.1% formic acid delivered to the electrospray emitter at 500 nL/min through the sheath liquid capillary. Unlabeled peptides were analyzed in data independent acquisition (DIA) mode using the UDMS^E^ method with instrument settings previously reported for this instrument, unless otherwise stated.^36,37^

### Hydrogen-Deuterium Exchange Sample Preparation and Workflow

For HDX–MS, stock solutions of either 2 mg/mL HPLC peptide standard, 4.4 mM ATII with 4.4 mM BK or 120 mg/mL intact bovine Hb were prepared in 50 mM ammonium bicarbonate in H_2_O, pH 6.79. An aliquot (13.75 μL) was diluted 20-fold in either 50 mM ammonium bicarbonate in H_2_O (pH 6.79; undeuterated control), 50 mM ammonium bicarbonate in D_2_O (pH_read_ 6.27), or 6 M urea in D_2_O (pH_read_ 3.04; maximally deuterated control). The peptide standards were incubated overnight and quenched by 20-fold dilution into ice cold 0.24% formic acid in 1/4 (v/v) ACN/H_2_O, which lowered the solution pH_read_ to 2.85. The intact Hb was exposed to the deuterated buffer for five timepoints (5, 50, 500, 5000, and 50,000 s). The temperature was maintained at 22 °C in an Eppendorf Thermomixer Compact (Eppendorf, Hamburg, Germany) for the three longest timepoints, and the deuteration reaction was quenched by preparing a 20-fold dilution of an aliquot of the labeled protein in ice cold 3.5 mg/mL pepsin with 1% formic acid in H_2_O, which lowered the solution pH_read_ to 2.31. A pulse-label control was prepared by incubating an aliquot of the stock Hb standard for 50,000 s in the thermomixer and then performing a 5 s labeling step, as described above. The protein/pepsin mixture was vortexed for ∼5 s and an aliquot placed in a PCR tube containing ice cold ACN, such that the final concentration of ACN was 25% (v/v). The PCR tube was removed from the ice and placed in a laboratory-designed, in-house machined acrylic sample pod featuring an o ring for a gas-tight seal and connected to a N_2_ (g) in-house line (Figure S7). The assembled sample pod was connected to the separation capillary inlet by passing the separation capillary inlet (Figure S8) through a union assembly with a tubing sleeve through a single ferrule (P-720; F-180; F-120, Idex Health & Science, Oak Harbor, WA) at the inlet side, and a sample plug was injected at 5 psi for 3 s. A 16 mm length of 2 mm OD, 1 mm ID PEEK tubing (6330N11, McMaster-Carr, Elmhurst, IL, USA) was secured with epoxy (36-2421, Biltema, Gothenburg, Sweden) to the junction of the tubing sleeve and the F-120 ferrule to provide a gas-tight fitting to the acrylic sample and BGE pod using a 2 mm push-in fitting (QSM-M5-2, Festo, Esslingen, Germany). When the pods are attached to the device, 1–2 mm of the capillary inlet will be submerged in the solution in the pods, and upon introduction of N_2_ (g) into the pods, solution will be forced into the capillary. After injection, the sample pod was removed, the capillary inlet rinsed with water (Figure S6) and replaced with another acrylic pod containing BGE in a stainless-steel vial connected to both N_2_ (g) and the HVPS (Figures S3–S5). A plug of BGE (20 psi, 3 s) was injected into the capillary to push the sample plug into the thermostatic region of the capillary. After the plug injection, the HVPS was ramped, and acquisition was started.

### Data Evaluation

Peptic peptides were sequenced with PLGS 3.0.3 (Waters Corporation, Manchester, United Kingdom), as previously described,^36,37^ with a modification of setting enzymes to “non-specific.” CE–MS data obtained with UDMS^E^ were searched against a database of UNIPROT peptide sequence entries for HBA_BOVIN, HBB_BOVIN, and PEPA_PIG. Complete data were further processed with the HX-DEAL module in Mass Spec Studio for HDX-analysis.^38^Tables S1–S5 show summaries of the experiments according to the Gothenburg format.

## Results and Discussion

After machining and assembly of the cooled fused-silica capillary platform (Figure 1), we assessed its performance and compared it with a previously published report on a microchip electrophoresis device.^16^ Here, we first present benchmarks of the CE platform with a focus on peak capacities for several separation conditions as well as the analysis of peptide standards for HDX to demonstrate acceptable deuterium back exchange. We then present the results of a bottom-up analysis of bovine Hb for five timepoints and two different separation conditions.

### Peak Capacity

Peak capacity is an important factor in maximizing the utility of generated data in HDX experiments. Peak capacity (*n*_c_) is defined as “the maximum number of peaks... [that can] be separated on a given column.”^39^ It is an entity that is dependent on the separation efficiency in both CE and LC. In CE, the capillaries have no packing, and thus, plate counts are not subject to Eddy diffusion as in LC. Hence, there is no mass transfer as the separation is based on the analyte’s behavior in an electric field. This leaves the common factor of diffusivity as the single major source of band broadening in CE separations. Recently, several *n*_c_ calculations appropriate for use with microchip electrophoresis and CE have been derived.^39^

In our analysis of *n*_c_ achieved by cold CE separations, we used the following equation describing the upper limit peak capacity
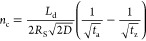
5where *R*_s_ is the resolution, *L*_d_ is the migration distance, *D* is the analyte’s diffusion coefficient, and *t*_a_ and *t*_z_ are the migration times of the first and last peaks of interest, respectively. Because the peak width in CE is migration time-dependent and nonlinear, increasing by the 3/2-power of migration time, in contrast to an assumed constant peak width in gradient chromatography, we believe that the *n*_c_ values for our CE separations are more appropriate than those reported using the traditional 4σ method common in LC. Supporting Information Table S6 shows calculated *n*_c_ for several different BGEs investigated at two different temperatures and field strengths for separations of labeled quenched peptide standards, including angiotensin II (ATII, analyte A in eq 5) and met-enkephalin (ME, analyte Z in eq 5). The initial separation of standards, as well as triplicate measures of deuterated, quenched, digested or intact Hb, on the LPA-coated capillary was performed using a 60 s voltage ramp by remotely controlling the HVPS with a digital potentiometer operated by an Arduino device. Only the −5 °C samples on LPA capillaries were automatically ramped. We found that optimal peak capacities were obtained at 0 °C with a BGE consisting of 1% FA, 25% ACN for BFS capillaries, and 10% HAc for LPA capillaries. The values of *n*_c_ for these conditions averaged 72 on BFS and 107 on LPA capillaries when calculated using the first (ATII) and last (ME) migrating standard peptides. When we subsequently measured peptic digests of Hb under these two conditions, the *n*_c_ averaged 120 on BFS and 175 on LPA capillaries. Thus, *n*_c_ for the LPA capillary exceeded that of the BFS capillary by almost 50%. The differences between the BFS and LPA capillaries are not surprising as the LPA coating has been used in electrophoresis since 1985 to eliminate solute adsorption and electroosmotic flow.^40^ In addition to the increased peak capacity of the LPA capillaries, the sequence coverage of Hb is increased due to the disruption of adsorption of hydrophilic peptides to the silanol groups of the capillary wall. The Kyte–Doolittle hydrophobicity indices of all identified peptides present in the final Hb data set have been mapped in a hydrophobicity histogram (Figure S9), and a clear trend of increased coverage of hydrophilic peptides is seen for the LPA capillaries.^41^ This is well in line with previous findings that more hydrophilic peptides are detected with CE versus reverse phase LC.^42^ We chose to use 40 μm ID capillaries as we have done so in our previous work.^34,43^ The use of larger ID capillaries at subzero temperatures has been reported to be feasible and with the result that more sample can be injected in a shorter plug length versus smaller lumen capillaries while attenuating the band broadening that typically accompanies sample injection on larger lumen capillaries.^33^

In comparison to the peak capacity values reported previously in a study involving both microchip electrophoresis (23 cm separation channel) and LC for HDX separations,^16^ we have obtained values that are placed inside of this range (Table 1). While it is difficult to make a high-quality comparison of the deuterium retention of our platform versus microchip CE, since we do not have access to the other study’s raw data and their demonstrations of deuterium uptake are displayed using only up to ∼50% of the *y*-axis of the plots (making precise visual readouts very difficult), we were able to compare the peak capacity of 0 °C CE to 0 °C LC and ambient microchip CE (Table 1). While our estimated upper peak capacity is less than that for the microchip electrophoresis method, our sequence coverage is higher. In comparison to the results obtained in the microchip electrophoresis study,^16^ our UDMS^E^ identifications found three of the six peptides reported in that paper. A very recent report using higher than normal LC flow rates has reported achieving peak capacities of 59.6 in a 10 min gradient using 200 μL/min flow rates,^15^ and while this is an improvement over the LC separation reported in Table 1, it is still below the capacity attainable on CE tested in this study and elsewhere.^16^

**Table 1:** Separation Characteristics for Bare Fused-Silica and Linear Polyacrylamide-Coated Capillaries Compared to LC and Microchip Electrophoresis Used for HDX–MS, Values Given as Coverage % (Redundancy)^a^

				coverage % (redundancy)			
method	separation time (min)	peak capacity@*r* = 1^b^	injected amount (fmol)	HBA	HBB	no. of HVPS	ESI flow rate (μL min^–1^)
0 °C LC^16^	7	31	50,000	97.9 (6.62)	92.4 (5.20)	0	100.00
ambient microchip electrophoresis^16^	1	351	5	48.2 (3.29)	84.8 (1.29)	5	0.63
0 °C CE (BFS)	3.5	120	50	75.4 (2.15)	79.3 (2.03)	1	0.50
0 °C CE (LPA)	8	175	50	99.3 (3.34)	95.9 (3.85)	1	0.50

a Abbreviations: BFS, bare fused silica; LPA, linear polyacrylamide; and HVPS, high voltage power supplies.

b Estimated with eq 5 for electrophoretic separations. Values for LC are given as previously reported estimates using the 4σ method.

### Back Exchange and Carryover

The most frequently used peptide standards in HDX–MS, bradykinin (BK) and ATII, comigrated on a BFS capillary with the BGE used in this study; therefore, we also performed analysis of a HPLC peptide standard mix. A crucial consideration in HDX experiments is the back exchange (BE) of deuterons under quench conditions and during the separation step. BE was calculated according to eq 6
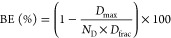
6where *D*_max_ is the deuterium content of the peptide standards, *N*_D_ is the number of labile backbone amide hydrogens of the peptides (calculated as the number of amino acids minus the number of prolines minus 1), and *D*_frac_ is the fraction of deuterium in the labeling buffer.

The peptide standards were also assessed for BE, and the results for BFS and LPA capillaries at 0 °C and using 20 kV as separation potential are listed in Table 2. After the first separation, where BE was absent, a fresh standard was prepared, labeled, quenched, and analyzed to confirm the validity of the original result. The result was replicated with the newly prepared standard. Because BK comigrates with ATII on BFS capillaries under the conditions investigated, it was not as thoroughly assessed as ATII or ME. BE values of 20% on BFS and 34% on LPA capillaries were achieved for ATII. The value of BE for ATII on LPA capillaries was similar to previously reported values from commercial and laboratory-modified UPLC platforms (28–36%), while on BFS capillaries, it approached BE levels achieved by direct infusion of a fully deuterated standard.^44,45^

**Table 2:** Deuterium Back Exchange Values for Standard Peptides Assessed with CE–MS at 0 °C and 20 kV, Values Given as Mean (% RSD), *n* = 3^a^

capillary coating	BGE	pH^b^	peptide	deuteron uptake	back exchange (%)
					
BFS	1% FA, 25% ACN	2.23	ATII	4.55 (0.65)	20 (2.6)
LPA	10% HAc	2.17		3.76 (0.97)	34 (1.9)
					
BFS	1% FA, 25% ACN	2.23	BK	3.76 (0.27)	21 (1.0)
LPA	10% HAc	2.17			
					
BFS	1% FA, 25% ACN	2.23	ME	4.74 (1.7)	0
LPA	10% HAc	2.17		1.2 (27)	68 (13)

a Abbreviations: ACN, acetonitrile; ATII, angiotensin II; BGE, background electrolyte; BFS, bare fused silica; BK, bradykinin; FA, formic acid; HAc, acetic acid; LPA, linear polyacrylamide; and ME, Met-enkephalin.

b Measured at 20 °C.

While ME is not traditionally used to assess BE in HDX–MS experiments, it seemed appropriate to include it in the analysis because it was the slowest migrating peptide in the HPLC peptide standard, as well as the most hydrophobic peptide in the mixture. Three other peptides in the standard mixture were not assessed for BE: gly–tyr, and val–tyr–val as they were much shorter than any typical peptide used for structural analysis; leu-enk was not assessed as it was present in the sheath liquid for use as lock mass correction. While the chosen BGE composition was based on the minimum BE achieved for peptide standards, this does not necessarily mean the BGEs were the best choice for achieving maximum sequence coverage or peak capacity. During development, much time was spent on analyzing the DMF-containing BGE and BFS capillaries as these were able to separate and detect 10 of 11 peptides from an HPLC peptide retention time marker standard mixture. Although the aprotic-modified BGEs showed promise for peptide mapping,^46^ further work is needed to assess the viability of such an approach with the short capillaries and low temperatures used in our HDX workflow. Improved sensitivity and thus sequence coverage can also be obtained for CE-based proteomics platforms, as demonstrated by trace-sensitive analyses by the Nemes group, achieving amol to zmol sensitivity for peptides.^47,48^

Assessing deuterium BE required the manual inspection of summed mass spectra to obtain deuteron uptake values. Spectral signal from peptides which have been retained in the system become problematic because as they sit in the protonated solvent of the separation modality, deuterons will exchange before the material ultimately reaches the detector. There has been much effort to address this phenomenon in the HDX community.^17,18^ During data analysis, we have not observed any signs of carryover in the inspected mass spectra following injections of standard peptides, intact Hb, or peptic digests of Hb. Sample carryover was assessed by extracting ion electropherograms for the 18 most abundant identified peptic peptides of Hb from standard peptide separations injected after peptic digests of Hb. The two most hydrophobic and the two most hydrophilic Hb peptides according to their Kyte–Doolittle hydrophobicity indices were also searched for as carryover in the following separations. The use of a peptide standard containing two or five peptides in the assessment of carryover is justified due to the fact that a failed injection can result in not seeing any signal from the analytes being injected, and therefore, the analysis of a successful blank injection could be indistinguishable from a failed blank injection. Observing peaks from the peptide standards demonstrates via peak shape and migration time, that the injection was successful and the capillary is performing normally. Since in CE we would only expect carryover from residual material on the outside of the capillary, ensuring that the capillary inlet has been immersed into the sample gives us the highest probability of observing carryover. An example of the lack of observable carryover in an HPLC peptide standard separation following a standard capillary conditioning step (10 min 20 psi flushing with 10% HAc BGE) after the injection of the peptic digests of a quenched 5 s labeling timepoint is shown in Table S7. For the assessment of carryover, the undeuterated peptide ion base peak *m*/*z* was extracted as an EIE ± 0.02 Da, as well as the *m*/*z* for every isotopologue expected between the base peak of the deuterated peptide ion and the base peak of the undeuterated peptide ion. We searched data for carryover in such a manner on both BFS and LPA capillaries operated at −5, 0, 2 °C, and ambient temperature for separations using 5% HAc; 10% HAc; 20% DMF, 20% HAc; 10% DMF, 10% ACN, 20% HAc; and 25% ACN, 1% FA. Carryover from HPLC peptide standards (*n* = 14); a standard of ATII & BK (*n* = 7); intact Hb (*n* = 1); and peptic digests of Hb (*n* = 9) was searched for in the data file from the immediately following separation after one sequence of capillary conditioning. Additionally, during curation of the Hb data sets, more than 1400 spectra were inspected during workup in Mass Spec Studio. We found no legitimate instances of carryover in these data.

This finding is not surprising based on the low amount of sample injected into the capillary versus the amount of sample used for LC (tens of fmol vs tens of pmol), and it has been suggested previously that CE separations have the potential to overcome the issue of carryover.^28^ Between sample injections, the replenishing of BGE by flushing multiple capillary volumes of fresh BGE for LPA capillaries and the extended NaOH surface cleaning for BFS capillaries appears sufficient to prevent any carryover signal from being observed.

### Bovine Hemoglobin

**Figure 2:**
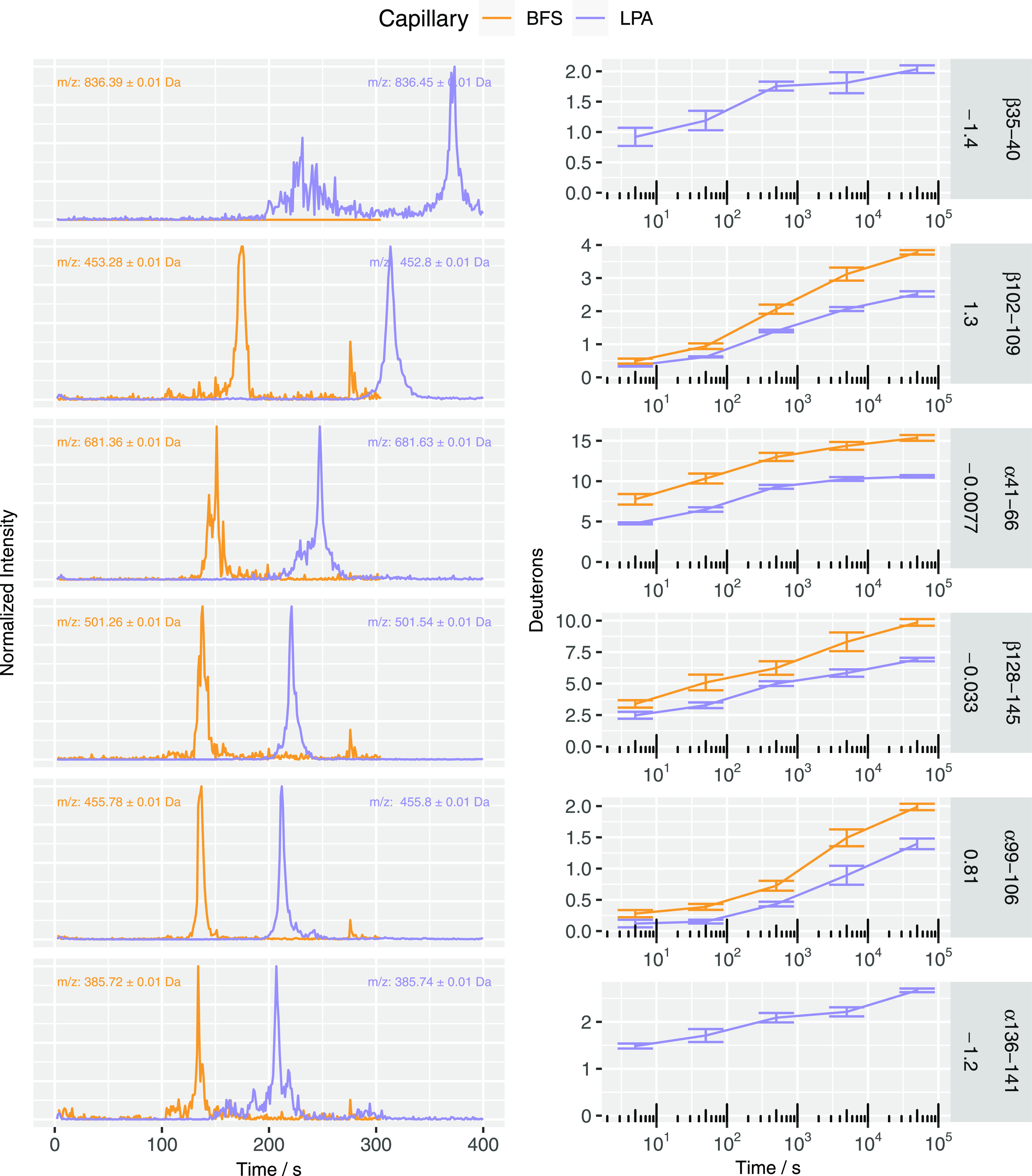
Intensity-normalized extracted ion electropherograms demonstrate differences in migration times between BFS and LPA capillaries and deuterium uptake plots for six representative peptides from peptic digests of labeled Hb. The orange trace shows the results from separations using a BFS capillary, and the purple trace shows the results from separations using an LPA-coated capillary. Annotation of the peptide sequence and their corresponding Kyte–Doolittle hydrophobicity index are provided on the right borders of each panel row. (Left) Representative peak shapes and migration times are shown at the 500 s labeling timepoint. (Right) Deuterium uptake was found to be more retained with bare-fused silica capillaries. Error bars represent one standard deviation, *n* = 3 per timepoint. However, some peptides were only present in sufficient abundance across all incubation times with the LPA coating, as shown by the absence of the orange trace for BFS capillaries in two of the six panels above. α136–141 is shown at the 500 s timepoint for this particular sample separated on a BFS capillary, but it was not of sufficient quality at later timepoints and was omitted from the final data set, and as a result, the deuterium uptake plots do not include this peptide. β35–40 was not detected at all and could not be included in deuterium uptake plots.

After selecting the best BGE composition for each of the capillary surfaces, deuterium labeling at five timepoints was carried out. Table 3 shows peptic peptides identified by UDMS^E^ analysis and used for the full HDX time course, whereas Table 4 shows the peak width and migration time reproducibility for the different separation conditions for six peptides chosen based on their Kyte–Doolittle hydrophobicity indices. Figure 2 shows their corresponding electropherograms and deuterium uptake. In some cases, for the sake of providing an uncluttered graphical representation, the ion from the extracted ion electropherogram is not the base peak ion for that peptide due to an isobaric ion showing up in the trace at a different migration time. It was observed that peak shapes were more symmetric, and the signal intensity was around an order of magnitude higher for the peptides separated on LPA capillaries than that on BFS capillaries. Although LPA-coated capillaries provided an overall lower deuterium retention in terms of absolute values when compared to BFS capillaries (Table 2 and Figure 2), we did not detect any differences in the deuterium uptake rates (Figure S10).

**Table 3:** Protein Coverage of Peptic Peptides of Hemoglobin Suitable for HDX Analysis with CE–MS^a^

capillary coating	BGE	pH^b^	protein	% coverage	no. of peptides	average peptide length (#AA)	redundancy
BFS	1% FA,25% ACN	2.23	α-Hb	75.4	13	24	2.15
			β-Hb	79.3	17	18	2.03
LPA	10% HAc	2.17	α-Hb	99.3	22	23	3.34
			β-Hb	95.9	30	19	3.85

a Abbreviations: ACN, acetonitrile; BGE, background electrolyte; BFS, bare fused silica; FA, formic acid; HAc, acetic acid; and LPA, linear polyacrylamide.

b Measured at 20 °C.

**Table 4:** Separation Performance for Six Representative Hb Peptic Peptides Measured across All HDX Timepoints on BFS (*n* = 15 Injections) and LPA (*n* = 15 Injections) Capillaries, Values Given as Mean (% RSD)^a^

	peptide	hydrophobicity^b^	capillary coating	migration time (s)	FWHM (s)
	α 41–66	0.008	BFS	148 (3.8)	11.4 (29)
			LPA	250 (2.5)	9 (22)
	α 99–106	0.813	BFS	137 (4.5)	6.9 (20)
			LPA	212 (2.1)	6.7 (15)
	α 136–141	–1.23	BFS	N/A	N/A
			LPA	207 (2)	8.3 (24)
	β 35–40	–1.40	BFS	N/A	N/A
			LPA	384 (3.4)	13.5 (23)
	β 102–109	1.33	BFS	177 (6.5)	8.9 (18)
			LPA	321 (3.1)	11 (22)
	β 128–145	–0.033	BFS	139 (4.6)	7.3 (28)
			LPA	222 (2.1)	7.8 (22)

a Abbreviations: BFS, bare fused silica; and LPA, linear polyacrylamide.

b Kyte–Doolittle hydrophobicity index.

The higher migration time RSDs observed for the BFS capillaries are likely due in part to the fact that collecting the triplicate timepoint measurements on BFS capillaries spanned more than three weeks and required multiple capillary replacements. The two most polar peptides in the list, α 136–141 and β 35–40, were detected in the BFS separations, but by the 500 s labeling timepoint, the signals were unusable. Since the migration times and peak widths were initially recorded during manual curation of these six peptides for the purpose of reporting D uptake (Figure 2), further monitoring of these two ions on BFS capillaries was halted.

Although BFS capillaries offered faster migration times, the analysis took longer than when using LPA capillaries because the BFS capillaries required more rinsing steps between sample injections. An additional consideration was residual NaOH left on the emitter after conditioning the BFS capillaries. Adding a tip-rinsing station to the platform, such as that used by the Sciex Digital Picoview nanospray ion source, would improve the robustness of this workflow. In contrast to the work on BFS that spanned a long time, measurement of the complete HDX time course on LPA was performed within an intense 21 h time period using a single capillary. While the separation reproducibility was notably better on the LPA capillary, it should be emphasized that these measurements were performed with manual voltage ramping. Incorporation of automated voltage ramping along with instrument triggering will likely result in better migration time reproducibility. Also, migration time alignment could be an option for these data sets to enable time savings during data curation. While the separations in this study were carried out on rather short capillaries, for complex mixtures and/or larger proteins where more resolving power is needed from the separation, the benefits of a longer capillary for increasing sequence coverage may be of added benefit. Furthermore, while it is uncommon that a sample may be available at 120 mg/mL concentration, we chose this concentration in line with the previous report for microchip electrophoresis.^16^ However, here we are performing a 400-fold dilution of the sample prior to injection while still injecting only ∼50 fmol of protein digest. This is ∼3 orders of magnitude less material than typical recent HDX workflows (10–50 pmol).^44,48−51^

**Figure 3:**
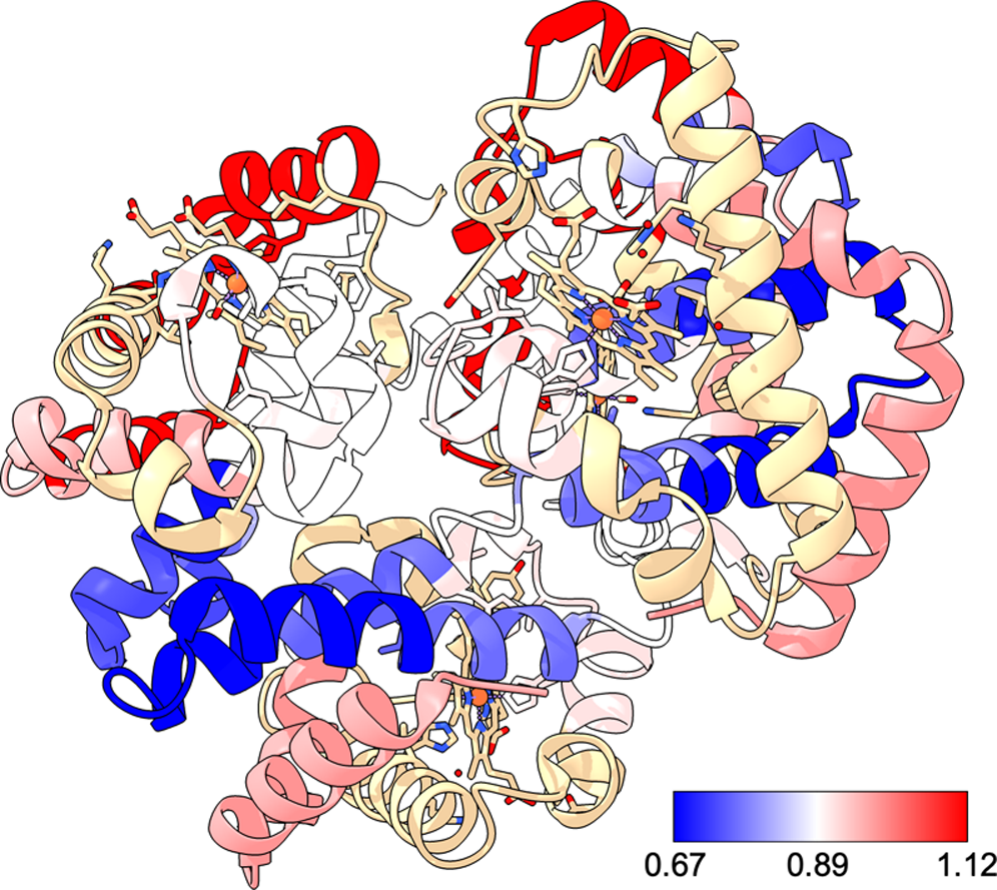
Overlay of HDX data for Hb on the PDB structure 1FSX. Hb labeled with D_2_O under native conditions was compared to that labeled after denaturation with 6 M urea. The color scale indicates the ratio of D uptake for native vs urea after 50,000 s of deuterium incorporation.

Although Hb is a well-studied protein due to its importance for the uptake of oxygen in the blood, we wanted to investigate how well our HDX CE–MS platform performed in terms of mapping structurally relevant information. Additionally, it is suggested to benchmark new HDX platforms using standard proteins such as bovine Hb.^52^ We compared the relative uptake of deuterium in Hb under native conditions with that after subjecting a sample of Hb to denaturing conditions with 6 M urea (Tables S1–S5, Figures S9 and S10). Figure 3 shows the relative protection of the different regions of the Hb tetramer. We noted that sites that were more protected from HDX in the native state coincided with the interaction sites for the Hb subunits.^53^ Specifically, the residues R32–Y43 and L92–D127 on α-Hb and R29–E42 and D98–Q130 on β-Hb coincide with where these two monomers bind to each other. Our data showed decreased susceptibility for HDX for the native state of Hb compared to the urea-exposed state in those regions (Figures 3 and S11) We consider this finding to validate our method as a promising tool for structural proteomics, enabling characterization of molecular binding and conformational dynamics, such as those encountered in protein–ligand interactions.

## Conclusions

We have provided proof of concept for low-temperature CE–MS applied to in-solution labeling HDX. Although BFS capillaries provide fast peptide separations and minimal loss of deuterium from labeled peptides, our findings show that LPA-coated capillaries are superior for HDX CE–MS. This rationale is based on the ability of LPA-coated capillaries to offer excellent peak capacity and largely improved sequence coverage while requiring less instrument time and agreeing with BFS experimental uptake rates. LPA capillaries are routinely used for intact protein analysis and also lend support for the application of low-temperature CE–MS for intact proteins to be fragmented by electron-capture dissociation or electron-transfer dissociation. Due to the nature of electrophoretic separations, we do not observe carryover. This happens due to the standard procedures for replenishing BGE by flushing the capillary with multiple volumes of BGE (or other solvents) between sample injections and because of the simplicity of flow path components. The beneficial lack of carryover with our system stands in stark contrast to LC-based HDX platforms, which require thorough assessment and time spent mitigating carryover. There remain many avenues to pursue for further optimization of this platform, including, but not limited to, BGE optimization (pH, organic content, and concentration), a concentrating/desalting step, immobilized/embedded protease digestion, upgrading the Peltier element to allow even colder separations, incorporating a sheathless electrospray interface, alternate capillary coatings, and assessing longer or shorter capillaries. Additional investigations into the tolerance of the separation for salts and solutes common in protein chemistry will also be a focus of future optimization. Several compounds such as the reducing agent tris(2-carboxyethyl)phosphine and anionic lipids or detergents which prove detrimental to LC separations should migrate away from the mass spectrometer under the electrophoretic conditions employed here, and their impact on the CE separations will occur in future optimizations.

## Data Availability

The mass spectrometry proteomics data have been deposited to the ProteomeXchange Consortium (http://proteomecentral.proteomexchange.org) via the PRIDE partner repository with the DOI 10.6019/PXD034245.

## Supporting Information Available

Supplementary Table S1: Experimental conditions for HDX CE-MS; S2–S5: HDX uptake for Bovine hemoglobin; S6: peak capacities for different separation conditions; S7: Carryover assessment; Supplementary Figure S1: Ohm's plot; S2: Schematic of pneumatic system; S3–S8 Diagrams of connections and fittings; S9: Hydrophobicity histogram; S10 Deuterium uptake plots for Bovine hemoglobin; S11 Relative protection plot (PDF)
