# Supplementary material for: Zero-Degree Celsius Capillary Electrophoresis Electrospray Ionization for Hydrogen Exchange Mass Spectrometry
Source: Anal Chem. 2022 Dec 22;95(2):1149–58. doi: 10.1021/acs.analchem.2c03893 (PMC9850406; doi:10.1021/acs.analchem.2c03893)
Supplement: Supplementary file 1 — ac2c03893_si_001.pdf [file ac2c03893_si_001.pdf]

Supporting Information: Zero-Degree Celsius Capillary Electrophoresis Electrospray Ionization for Hydrogen Exchange Mass Spectrometry

Jordan T. Aerts,[†] Per E. Andrén,[†,‡] and Erik T. Jansson[*,†]

†*Department of Pharmaceutical Biosciences, Uppsala University, 751 24 Uppsala, Sweden*

‡*Science for Life Laboratory, Spatial Mass Spectrometry, Uppsala University, 751 24 Uppsala, Sweden*

E-mail: erik.jansson@farmbio.uu.se

List of Tables

S1 Summary of experimental conditions for HDX CE–MS of bovine hemoglobin. S-4

S2 HDX uptake for HBA_BOVIN with bare fused-silica capillaries. S-5

S3 HDX uptake for HBB_BOVIN with bare fused-silica capillaries. S-8

S4 HDX uptake for HBA_BOVIN with linear polyacrylamide coated fused-silica capillaries. S-11

S5 HDX uptake for HBB_BOVIN with linear polyacrylamide coated fused-silica capillaries. S-15

S6 Separation characteristics for bare fused-silica and linear polyacrylamide coated capillaries S-20

S7 Assessment of carryover S-21

List of Figures

S1 Ohm's plot for different capillary coatings and temperatures. S-22

S2 Schematic of pneumatic system used for performing capillary conditioning and sample injection S-23

S3 Diagram of major connections and fittings used for CE S-24

S4 Image showing device with cover removed to highlight important components. S-25

S5 Close-up section representation of fittings used for gas- and electronic connections to BGE pod. S-26

S6 Capillary inlet rinsing to prevent carryover S-27

S7 Sample injection pod connected to gas manifold S-28

S8 Section representation of separation capillary inlet assembly. S-29

S9 Hydrophobicity histogram for HDX suitable Hb peptides S-30

S10 Deuterium uptake plots for bovine hemoglobin S-31

S11 Relative protection plot S-32

Table S1: Summary of experimental conditions for HDX CE–MS of bovine hemoglobin.

	LPA		BFS	
HDX reaction details	HBA	HBB	HBA	HBB
HDX time course (min)	0, 0.0833, 0.833, 8.33, 83.33, 833.33			
Replicates (technical)	3		3	
Control sample conditions	6M Urea 833.33 min			
Control sample replicates (technical)	1		2	
Back-exchange (mean)	52.33%	46.45%	37.11%	25.31%
# of Peptides	21	28	12	17
Sequence coverage	99.30%	95.17%	74.65%	77.93%
Average peptide length	22.6	19.5	24.4	18.2
Redundancy	3.34	3.84	2.15	2.03
Repeatability (av. RSD)	0.035	0.040	0.030	0.038

Table S2: HDX uptake for HBA_BOVIN with bare fused-silica capillaries.

Protein state	StaMT	End	Sequence	Charge	Peptide mass (Da)	MT (min)	HDX time (min)	Uptake (D)	Uptake SD (D)
6M urea	110	136	ASHLPSDFTPAVHASLDKFLANVSTVL	4	2836.48	2.67	833.33	14.21	0.03
Native	110	136	ASHLPSDFTPAVHASLDKFLANVSTVL	4	2836.48	2.67	833.33	9.80	0.14
Native	110	136	ASHLPSDFTPAVHASLDKFLANVSTVL	4	2836.48	2.67	83.33	6.70	0.36
Native	110	136	ASHLPSDFTPAVHASLDKFLANVSTVL	4	2836.48	2.67	8.33	4.90	0.24
Native	110	136	ASHLPSDFTPAVHASLDKFLANVSTVL	4	2836.48	2.67	0.83	3.60	0.18
Native	110	136	ASHLPSDFTPAVHASLDKFLANVSTVL	4	2836.48	2.67	0.08	2.94	0.09
6M urea	110	137	ASHLPSDFTPAVHASLDKFLANVSTVLT	4	2937.52	2.70	833.33	15.02	0.04
Native	110	137	ASHLPSDFTPAVHASLDKFLANVSTVLT	4	2937.52	2.70	833.33	9.05	0.08
Native	110	137	ASHLPSDFTPAVHASLDKFLANVSTVLT	4	2937.52	2.70	83.33	6.60	0.36
Native	110	137	ASHLPSDFTPAVHASLDKFLANVSTVLT	4	2937.52	2.70	8.33	5.10	0.20
Native	110	137	ASHLPSDFTPAVHASLDKFLANVSTVLT	4	2937.52	2.70	0.83	3.70	0.10
Native	110	137	ASHLPSDFTPAVHASLDKFLANVSTVLT	4	2937.52	2.70	0.08	2.90	0.14
6M urea	33	46	FLSFPTTKTYFPHF	3	1731.87	2.57	833.33	6.74	0.05
Native	33	46	FLSFPTTKTYFPHF	3	1731.87	2.57	833.33	6.83	0.04
Native	33	46	FLSFPTTKTYFPHF	3	1731.87	2.57	83.33	6.20	0.17
Native	33	46	FLSFPTTKTYFPHF	3	1731.87	2.57	8.33	5.20	0.13
Native	33	46	FLSFPTTKTYFPHF	3	1731.87	2.57	0.83	4.08	0.02
Native	33	46	FLSFPTTKTYFPHF	3	1731.87	2.57	0.08	3.40	0.13
6M urea	99	106	KLLSHSLL	2	909.56	2.11	833.33	3.39	0.05
Native	99	106	KLLSHSLL	2	909.56	2.11	833.33	1.85	0.03
Native	99	106	KLLSHSLL	2	909.56	2.11	83.33	1.20	0.13
Native	99	106	KLLSHSLL	2	909.56	2.11	8.33	0.43	0.06
Native	99	106	KLLSHSLL	2	909.56	2.11	0.83	0.19	0.02
Native	99	106	KLLSHSLL	2	909.56	2.11	0.08	0.17	0.04
6M urea	129	141	LANVSTVLTSKYR	3	1450.81	2.45	833.33	8.25	0.05
Native	129	141	LANVSTVLTSKYR	3	1450.81	2.45	833.33	8.39	0.04
Native	129	141	LANVSTVLTSKYR	3	1450.81	2.45	83.33	6.30	0.23
Native	129	141	LANVSTVLTSKYR	3	1450.81	2.45	8.33	4.50	0.20
Native	129	141	LANVSTVLTSKYR	3	1450.81	2.45	0.83	3.90	0.07
Native	129	141	LANVSTVLTSKYR	3	1450.81	2.45	0.08	3.50	0.11
6M urea	84	106	SDLHAHKLRVDPVNFKLLSHSLL	5	2638.47	2.23	833.33	10.66	0.03
Native	84	106	SDLHAHKLRVDPVNFKLLSHSLL	5	2638.47	2.23	833.33	10.10	0.18
Native	84	106	SDLHAHKLRVDPVNFKLLSHSLL	5	2638.47	2.23	83.33	9.00	0.38
Native	84	106	SDLHAHKLRVDPVNFKLLSHSLL	5	2638.47	2.23	8.33	7.20	0.36
Native	84	106	SDLHAHKLRVDPVNFKLLSHSLL	5	2638.47	2.23	0.83	5.80	0.22
Native	84	106	SDLHAHKLRVDPVNFKLLSHSLL	5	2638.47	2.23	0.08	4.20	0.29

Continued on next page

Table S2 – *Continued from previous page*

Protein state	StaMT	End	Sequence	Charge	Peptide mass (Da)	MT (min)	HDX time (min)	Uptake (D)	Uptake SD (D)
6M urea	81	106	SELSDLHAHKLRVDPVNFKLLSHSLL	5	2967.63	2.23	833.33	13.00	0.13
Native	81	106	SELSDLHAHKLRVDPVNFKLLSHSLL	5	2967.63	2.23	833.33	12.50	0.26
Native	81	106	SELSDLHAHKLRVDPVNFKLLSHSLL	5	2967.63	2.23	83.33	11.40	0.29
Native	81	106	SELSDLHAHKLRVDPVNFKLLSHSLL	5	2967.63	2.23	8.33	9.70	0.26
Native	81	106	SELSDLHAHKLRVDPVNFKLLSHSLL	5	2967.63	2.23	0.83	8.10	0.25
Native	81	106	SELSDLHAHKLRVDPVNFKLLSHSLL	5	2967.63	2.23	0.08	6.70	0.27
6M urea	1	29	VLSAADKGNVKAAWGKVGGHAAEYGAEAL	4	2839.46	2.32	833.33	16.80	0.20
6M urea	1	29	VLSAADKGNVKAAWGKVGGHAAEYGAEAL	5	2839.46	2.32	833.33	16.50	0.07
Native	1	29	VLSAADKGNVKAAWGKVGGHAAEYGAEAL	4	2839.46	2.32	833.33	17.30	0.27
Native	1	29	VLSAADKGNVKAAWGKVGGHAAEYGAEAL	5	2839.46	2.32	833.33	17.20	0.10
Native	1	29	VLSAADKGNVKAAWGKVGGHAAEYGAEAL	4	2839.46	2.32	83.33	16.00	0.34
Native	1	29	VLSAADKGNVKAAWGKVGGHAAEYGAEAL	5	2839.46	2.32	83.33	15.80	0.26
Native	1	29	VLSAADKGNVKAAWGKVGGHAAEYGAEAL	4	2839.46	2.32	8.33	13.80	0.47
Native	1	29	VLSAADKGNVKAAWGKVGGHAAEYGAEAL	5	2839.46	2.32	8.33	13.60	0.63
Native	1	29	VLSAADKGNVKAAWGKVGGHAAEYGAEAL	4	2839.46	2.32	0.83	10.90	0.63
Native	1	29	VLSAADKGNVKAAWGKVGGHAAEYGAEAL	5	2839.46	2.32	0.83	10.90	0.61
Native	1	29	VLSAADKGNVKAAWGKVGGHAAEYGAEAL	4	2839.46	2.32	0.08	8.40	0.38
Native	1	29	VLSAADKGNVKAAWGKVGGHAAEYGAEAL	5	2839.46	2.32	0.08	8.20	0.47
6M urea	1	33	VLSAADKGNVKAAWGKVGGHAAEYGAEALERMF	5	3402.71	2.23	833.33	19.20	0.56
Native	1	33	VLSAADKGNVKAAWGKVGGHAAEYGAEALERMF	5	3402.71	2.23	833.33	17.30	0.31
Native	1	33	VLSAADKGNVKAAWGKVGGHAAEYGAEALERMF	5	3402.71	2.23	83.33	15.80	0.51
Native	1	33	VLSAADKGNVKAAWGKVGGHAAEYGAEALERMF	5	3402.71	2.23	8.33	13.80	0.77
Native	1	33	VLSAADKGNVKAAWGKVGGHAAEYGAEALERMF	5	3402.71	2.23	0.83	11.10	0.72
Native	1	33	VLSAADKGNVKAAWGKVGGHAAEYGAEALERMF	5	3402.71	2.23	0.08	8.40	0.56
6M urea	107	128	VTLASHLPSDFTPAVHASLDKF	4	2352.21	2.50	833.33	10.88	0.09
Native	107	128	VTLASHLPSDFTPAVHASLDKF	4	2352.21	2.50	833.33	6.30	0.12
Native	107	128	VTLASHLPSDFTPAVHASLDKF	4	2352.21	2.50	83.33	5.10	0.11
Native	107	128	VTLASHLPSDFTPAVHASLDKF	4	2352.21	2.50	8.33	4.40	0.27
Native	107	128	VTLASHLPSDFTPAVHASLDKF	4	2352.21	2.50	0.83	3.50	0.15
Native	107	128	VTLASHLPSDFTPAVHASLDKF	4	2352.21	2.50	0.08	2.70	0.16
6M urea	107	136	VTLASHLPSDFTPAVHASLDKFLANVSTVL	4	3149.68	2.75	833.33	16.40	0.15
Native	107	136	VTLASHLPSDFTPAVHASLDKFLANVSTVL	4	3149.68	2.75	833.33	10.70	0.13
Native	107	136	VTLASHLPSDFTPAVHASLDKFLANVSTVL	4	3149.68	2.75	83.33	7.30	0.39
Native	107	136	VTLASHLPSDFTPAVHASLDKFLANVSTVL	4	3149.68	2.75	8.33	5.20	0.22
Native	107	136	VTLASHLPSDFTPAVHASLDKFLANVSTVL	4	3149.68	2.75	0.83	3.60	0.14
Native	107	136	VTLASHLPSDFTPAVHASLDKFLANVSTVL	4	3149.68	2.75	0.08	2.87	0.07
6M urea	107	141	VTLASHLPSDFTPAVHASLDKFLANVSTVLTSKYR	5	3785.01	2.40	833.33	20.40	0.28
Native	107	141	VTLASHLPSDFTPAVHASLDKFLANVSTVLTSKYR	5	3785.01	2.40	833.33	15.01	0.06

Continued on next page

Table S2 – *Continued from previous page*

Protein state	StaMT	End	Sequence	Charge	Peptide mass (Da)	MT (min)	HDX time (min)	Uptake (D)	Uptake SD (D)
Native	107	141	VTLASHLPSDFTPAVHASLDKFLANVSTVLTSKYR	5	3785.01	2.40	83.33	11.20	0.56
Native	107	141	VTLASHLPSDFTPAVHASLDKFLANVSTVLTSKYR	5	3785.01	2.40	8.33	8.80	0.34
Native	107	141	VTLASHLPSDFTPAVHASLDKFLANVSTVLTSKYR	5	3785.01	2.40	0.83	7.10	0.10
Native	107	141	VTLASHLPSDFTPAVHASLDKFLANVSTVLTSKYR	5	3785.01	2.40	0.08	6.10	0.11

Table S3: HDX uptake for HBB_BOVIN with bare fused-silica capillaries.

Protein state	StaMT	End	Sequence	Charge	Peptide mass (Da)	MT (min)	HDX time (min)	Uptake (D)	Uptake SD (D)
6M urea	114	145	ARNFGKEFTPVLQADFQKVVAGVANALAHRYH	6	3553.87	2.32	833.33	19.60	0.01
Native	114	145	ARNFGKEFTPVLQADFQKVVAGVANALAHRYH	6	3553.87	2.32	833.33	17.60	0.17
Native	114	145	ARNFGKEFTPVLQADFQKVVAGVANALAHRYH	6	3553.87	2.32	83.33	14.40	0.98
Native	114	145	ARNFGKEFTPVLQADFQKVVAGVANALAHRYH	6	3553.87	2.32	8.33	10.00	1.10
Native	114	145	ARNFGKEFTPVLQADFQKVVAGVANALAHRYH	6	3553.87	2.32	0.83	7.10	0.51
Native	114	145	ARNFGKEFTPVLQADFQKVVAGVANALAHRYH	6	3553.87	2.32	0.08	5.70	0.19
6M urea	128	145	DFQKVVAGVANALAHRYH	4	1995.04	2.11	833.33	10.30	0.21
Native	128	145	DFQKVVAGVANALAHRYH	4	1995.04	2.11	833.33	10.00	0.12
Native	128	145	DFQKVVAGVANALAHRYH	4	1995.04	2.11	83.33	8.60	0.49
Native	128	145	DFQKVVAGVANALAHRYH	4	1995.04	2.11	8.33	6.20	0.97
Native	128	145	DFQKVVAGVANALAHRYH	4	1995.04	2.11	0.83	4.20	0.43
Native	128	145	DFQKVVAGVANALAHRYH	4	1995.04	2.11	0.08	3.27	0.06
6M urea	102	109	FKLLGNVL	2	902.56	2.65	833.33	4.05	0.02
Native	102	109	FKLLGNVL	2	902.56	2.65	833.33	3.74	0.01
Native	102	109	FKLLGNVL	2	902.56	2.65	83.33	3.10	0.22
Native	102	109	FKLLGNVL	2	902.56	2.65	8.33	1.80	0.17
Native	102	109	FKLLGNVL	2	902.56	2.65	0.83	0.70	0.17
Native	102	109	FKLLGNVL	2	902.56	2.65	0.08	0.37	0.07
6M urea	129	145	FQKVVAGVANALAHRYH	4	1880.02	2.06	833.33	9.72	0.09
Native	129	145	FQKVVAGVANALAHRYH	4	1880.02	2.06	833.33	10.00	0.21
Native	129	145	FQKVVAGVANALAHRYH	4	1880.02	2.06	83.33	8.70	0.47
Native	129	145	FQKVVAGVANALAHRYH	4	1880.02	2.06	8.33	5.90	0.96
Native	129	145	FQKVVAGVANALAHRYH	4	1880.02	2.06	0.83	3.90	0.28
Native	129	145	FQKVVAGVANALAHRYH	4	1880.02	2.06	0.08	3.20	0.17
6M urea	31	40	LVVYPWTQRF	2	1307.70	2.89	833.33	5.71	0.05
Native	31	40	LVVYPWTQRF	2	1307.70	2.89	833.33	4.64	0.03
Native	31	40	LVVYPWTQRF	2	1307.70	2.89	83.33	4.20	0.19
Native	31	40	LVVYPWTQRF	2	1307.70	2.89	8.33	3.50	0.16
Native	31	40	LVVYPWTQRF	2	1307.70	2.89	0.83	2.70	0.13
Native	31	40	LVVYPWTQRF	2	1307.70	2.89	0.08	2.00	0.20
6M urea	31	44	LVVYPWTQRFFESF	2	1817.91	3.13	833.33	8.24	0.02
Native	31	44	LVVYPWTQRFFESF	2	1817.91	3.13	833.33	7.60	0.20
Native	31	44	LVVYPWTQRFFESF	2	1817.91	3.13	83.33	6.80	0.32
Native	31	44	LVVYPWTQRFFESF	2	1817.91	3.13	8.33	6.00	0.19
Native	31	44	LVVYPWTQRFFESF	2	1817.91	3.13	0.83	5.00	0.11
Native	31	44	LVVYPWTQRFFESF	2	1817.91	3.13	0.08	4.32	0.10

Continued on next page

Table S3 – *Continued from previous page*

Protein state	StaMT	End	Sequence	Charge	Peptide mass (Da)	MT (min)	HDX time (min)	Uptake (D)	Uptake SD (D)
6M urea	31	47	LVVYPWTQRFFESFGDL	3	2103.05	3.23	833.33	9.70	0.16
Native	31	47	LVVYPWTQRFFESFGDL	3	2103.05	3.23	833.33	9.30	0.18
Native	31	47	LVVYPWTQRFFESFGDL	3	2103.05	3.23	83.33	8.20	0.42
Native	31	47	LVVYPWTQRFFESFGDL	3	2103.05	3.23	8.33	7.20	0.60
Native	31	47	LVVYPWTQRFFESFGDL	3	2103.05	3.23	0.83	6.55	0.10
Native	31	47	LVVYPWTQRFFESFGDL	3	2103.05	3.23	0.08	5.80	0.33
6M urea	1	13	MLTAEEKAAVTAF	2	1380.70	2.97	833.33	7.71	0.08
Native	1	13	MLTAEEKAAVTAF	2	1380.70	2.97	833.33	9.00	0.11
Native	1	13	MLTAEEKAAVTAF	2	1380.70	2.97	83.33	9.00	0.16
Native	1	13	MLTAEEKAAVTAF	2	1380.70	2.97	8.33	8.80	0.18
Native	1	13	MLTAEEKAAVTAF	2	1380.70	2.97	0.83	7.60	0.56
Native	1	13	MLTAEEKAAVTAF	2	1380.70	2.97	0.08	5.50	0.73
6M urea	1	30	MLTAEEKAAVTAFWGKVKVDEVGGEALGRL	5	3174.67	2.32	833.33	19.50	0.10
Native	1	30	MLTAEEKAAVTAFWGKVKVDEVGGEALGRL	5	3174.67	2.32	833.33	20.61	0.06
Native	1	30	MLTAEEKAAVTAFWGKVKVDEVGGEALGRL	5	3174.67	2.32	83.33	19.10	0.27
Native	1	30	MLTAEEKAAVTAFWGKVKVDEVGGEALGRL	5	3174.67	2.32	8.33	18.20	0.43
Native	1	30	MLTAEEKAAVTAFWGKVKVDEVGGEALGRL	5	3174.67	2.32	0.83	16.00	1.10
Native	1	30	MLTAEEKAAVTAFWGKVKVDEVGGEALGRL	5	3174.67	2.32	0.08	12.00	1.20
6M urea	72	95	NGMKHLDDLKGTFAALSELHCDKL	4	2655.31	2.32	833.33	15.11	0.09
Native	72	95	NGMKHLDDLKGTFAALSELHCDKL	4	2655.31	2.32	833.33	16.70	0.46
Native	72	95	NGMKHLDDLKGTFAALSELHCDKL	4	2655.31	2.32	83.33	15.60	0.29
Native	72	95	NGMKHLDDLKGTFAALSELHCDKL	4	2655.31	2.32	8.33	13.60	0.48
Native	72	95	NGMKHLDDLKGTFAALSELHCDKL	4	2655.31	2.32	0.83	10.70	0.58
Native	72	95	NGMKHLDDLKGTFAALSELHCDKL	4	2655.31	2.32	0.08	8.20	0.44
6M urea	126	145	QADFQKVVAGVANALAHRYH	4	2194.14	2.13	833.33	11.80	0.14
Native	126	145	QADFQKVVAGVANALAHRYH	4	2194.14	2.13	833.33	10.60	0.19
Native	126	145	QADFQKVVAGVANALAHRYH	4	2194.14	2.13	83.33	9.00	0.56
Native	126	145	QADFQKVVAGVANALAHRYH	4	2194.14	2.13	8.33	5.80	0.47
Native	126	145	QADFQKVVAGVANALAHRYH	4	2194.14	2.13	0.83	4.00	0.36
Native	126	145	QADFQKVVAGVANALAHRYH	4	2194.14	2.13	0.08	3.20	0.13
6M urea	130	145	QKVVAGVANALAHRYH	4	1732.95	2.01	833.33	9.08	0.09
Native	130	145	QKVVAGVANALAHRYH	4	1732.95	2.01	833.33	8.90	0.18
Native	130	145	QKVVAGVANALAHRYH	4	1732.95	2.01	83.33	7.90	0.38
Native	130	145	QKVVAGVANALAHRYH	4	1732.95	2.01	8.33	5.00	1.00
Native	130	145	QKVVAGVANALAHRYH	4	1732.95	2.01	0.83	3.80	0.21
Native	130	145	QKVVAGVANALAHRYH	4	1732.95	2.01	0.08	3.00	0.37
6M urea	14	30	WGKVKVDEVGGEALGRL	3	1811.99	2.23	833.33	9.40	0.17
Native	14	30	WGKVKVDEVGGEALGRL	3	1811.99	2.23	833.33	8.47	0.06

Continued on next page

Table S3 – *Continued from previous page*

Protein state	StaMT	End	Sequence	Charge	Peptide mass (Da)	MT (min)	HDX time (min)	Uptake (D)	Uptake SD (D)
Native	14	30	WGKVKVDEVGGEALGRL	3	1811.99	2.23	83.33	7.50	0.22
Native	14	30	WGKVKVDEVGGEALGRL	3	1811.99	2.23	8.33	7.10	0.29
Native	14	30	WGKVKVDEVGGEALGRL	3	1811.99	2.23	0.83	6.20	0.14
Native	14	30	WGKVKVDEVGGEALGRL	3	1811.99	2.23	0.08	5.20	0.27
6M urea	110	125	VVVLARNFGKEFTPVL	3	1788.03	2.64	833.33	9.20	0.13
Native	110	125	VVVLARNFGKEFTPVL	3	1788.03	2.64	833.33	6.86	0.10
Native	110	125	VVVLARNFGKEFTPVL	3	1788.03	2.64	83.33	6.00	0.14
Native	110	125	VVVLARNFGKEFTPVL	3	1788.03	2.64	8.33	4.90	0.42
Native	110	125	VVVLARNFGKEFTPVL	3	1788.03	2.64	0.83	3.70	0.16
Native	110	125	VVVLARNFGKEFTPVL	3	1788.03	2.64	0.08	2.80	0.28
6M urea	110	127	VVVLARNFGKEFTPVLQA	3	1987.13	2.75	833.33	9.40	0.67
Native	110	127	VVVLARNFGKEFTPVLQA	3	1987.13	2.75	833.33	6.85	0.08
Native	110	127	VVVLARNFGKEFTPVLQA	3	1987.13	2.75	83.33	6.10	0.19
Native	110	127	VVVLARNFGKEFTPVLQA	3	1987.13	2.75	8.33	5.20	0.36
Native	110	127	VVVLARNFGKEFTPVLQA	3	1987.13	2.75	0.83	3.70	0.16
Native	110	127	VVVLARNFGKEFTPVLQA	3	1987.13	2.75	0.08	2.80	0.20
6M urea	110	128	VVVLARNFGKEFTPVLQAD	3	2102.15	2.81	833.33	11.03	0.04
Native	110	128	VVVLARNFGKEFTPVLQAD	3	2102.15	2.81	833.33	7.57	0.05
Native	110	128	VVVLARNFGKEFTPVLQAD	3	2102.15	2.81	83.33	6.60	0.22
Native	110	128	VVVLARNFGKEFTPVLQAD	3	2102.15	2.81	8.33	5.20	0.42
Native	110	128	VVVLARNFGKEFTPVLQAD	3	2102.15	2.81	0.83	3.70	0.16
Native	110	128	VVVLARNFGKEFTPVLQAD	3	2102.15	2.81	0.08	2.80	0.22
6M urea	110	129	VVVLARNFGKEFTPVLQADF	3	2249.22	2.81	833.33	11.70	0.11
Native	110	129	VVVLARNFGKEFTPVLQADF	3	2249.22	2.81	833.33	7.50	0.07
Native	110	129	VVVLARNFGKEFTPVLQADF	3	2249.22	2.81	83.33	6.60	0.21
Native	110	129	VVVLARNFGKEFTPVLQADF	3	2249.22	2.81	8.33	5.20	0.43
Native	110	129	VVVLARNFGKEFTPVLQADF	3	2249.22	2.81	0.83	3.70	0.21
Native	110	129	VVVLARNFGKEFTPVLQADF	3	2249.22	2.81	0.08	2.90	0.16

Table S4: HDX uptake for HBA_BOVIN with linear polyacrylamide coated fused-silica capillaries.

Protein state	StaMT	End	Sequence	Charge	Peptide mass (Da)	MT (min)	HDX time (min)	Uptake (D)	Uptake SD (D)
6M urea	28	33	ALERMF	2	765.38	4.78	833.33	1.66	0.00
Native	28	33	ALERMF	2	765.38	4.78	833.33	0.48	0.05
Native	28	33	ALERMF	2	765.38	4.78	83.33	0.51	0.08
Native	28	33	ALERMF	2	765.38	4.78	8.33	0.40	0.04
Native	28	33	ALERMF	2	765.38	4.78	0.83	0.40	0.05
Native	28	33	ALERMF	2	765.38	4.78	0.08	0.42	0.06
6M urea	110	136	ASHLPSDFTPAVHASLDKFLANVSTVL	4	2836.48	5.31	833.33	9.34	0.00
Native	110	136	ASHLPSDFTPAVHASLDKFLANVSTVL	4	2836.48	5.31	833.33	7.10	0.18
Native	110	136	ASHLPSDFTPAVHASLDKFLANVSTVL	4	2836.48	5.31	83.33	4.80	0.47
Native	110	136	ASHLPSDFTPAVHASLDKFLANVSTVL	4	2836.48	5.31	8.33	3.60	0.25
Native	110	136	ASHLPSDFTPAVHASLDKFLANVSTVL	4	2836.48	5.31	0.83	2.30	0.14
Native	110	136	ASHLPSDFTPAVHASLDKFLANVSTVL	4	2836.48	5.31	0.08	1.88	0.10
6M urea	110	137	ASHLPSDFTPAVHASLDKFLANVSTVLT	4	2937.52	5.51	833.33	9.76	0.00
Native	110	137	ASHLPSDFTPAVHASLDKFLANVSTVLT	4	2937.52	5.51	833.33	6.23	0.05
Native	110	137	ASHLPSDFTPAVHASLDKFLANVSTVLT	4	2937.52	5.51	83.33	4.60	0.29
Native	110	137	ASHLPSDFTPAVHASLDKFLANVSTVLT	4	2937.52	5.51	8.33	3.60	0.25
Native	110	137	ASHLPSDFTPAVHASLDKFLANVSTVLT	4	2937.52	5.51	0.83	2.30	0.19
Native	110	137	ASHLPSDFTPAVHASLDKFLANVSTVLT	4	2937.52	5.51	0.08	1.80	0.11
6M urea	30	36	ERMFLSF	2	928.45	5.29	833.33	2.02	0.00
Native	30	36	ERMFLSF	2	928.45	5.29	833.33	0.70	0.04
Native	30	36	ERMFLSF	2	928.45	5.29	83.33	0.41	0.03
Native	30	36	ERMFLSF	2	928.45	5.29	8.33	0.24	0.02
Native	30	36	ERMFLSF	2	928.45	5.29	0.83	0.19	0.01
Native	30	36	ERMFLSF	2	928.45	5.29	0.08	0.20	0.04
6M urea	33	46	FLSFPTTKTYFPHF	3	1731.87	5.14	833.33	4.23	0.00
Native	33	46	FLSFPTTKTYFPHF	3	1731.87	5.14	833.33	4.40	0.10
Native	33	46	FLSFPTTKTYFPHF	3	1731.87	5.14	83.33	4.20	0.24
Native	33	46	FLSFPTTKTYFPHF	3	1731.87	5.14	8.33	3.80	0.32
Native	33	46	FLSFPTTKTYFPHF	3	1731.87	5.14	0.83	2.50	0.24
Native	33	46	FLSFPTTKTYFPHF	3	1731.87	5.14	0.08	2.15	0.08
6M urea	87	106	HAHKLRVDPVNFKLLSHSLL	5	2323.33	2.99	833.33	7.36	0.00
Native	87	106	HAHKLRVDPVNFKLLSHSLL	5	2323.33	2.99	833.33	6.90	0.12
Native	87	106	HAHKLRVDPVNFKLLSHSLL	5	2323.33	2.99	83.33	6.10	0.28
Native	87	106	HAHKLRVDPVNFKLLSHSLL	5	2323.33	2.99	8.33	4.90	0.27
Native	87	106	HAHKLRVDPVNFKLLSHSLL	5	2323.33	2.99	0.83	3.50	0.13
Native	87	106	HAHKLRVDPVNFKLLSHSLL	5	2323.33	2.99	0.08	2.80	0.20

Continued on next page

Table S4 – *Continued from previous page*

Protein state	StaMT	End	Sequence	Charge	Peptide mass (Da)	MT (min)	HDX time (min)	Uptake (D)	Uptake SD (D)
6M urea	99	106	KLLSHSLL	2	909.56	3.45	833.33	2.54	0.00
Native	99	106	KLLSHSLL	2	909.56	3.45	833.33	1.38	0.09
Native	99	106	KLLSHSLL	2	909.56	3.45	83.33	0.75	0.03
Native	99	106	KLLSHSLL	2	909.56	3.45	8.33	0.28	0.01
Native	99	106	KLLSHSLL	2	909.56	3.45	0.83	0.11	0.02
Native	99	106	KLLSHSLL	2	909.56	3.45	0.08	0.09	0.02
6M urea	129	141	LANVSTVLTSKYR	3	1450.81	4.60	833.33	5.72	0.00
Native	129	141	LANVSTVLTSKYR	3	1450.81	4.60	833.33	5.85	0.04
Native	129	141	LANVSTVLTSKYR	3	1450.81	4.60	83.33	4.70	0.24
Native	129	141	LANVSTVLTSKYR	3	1450.81	4.60	8.33	3.59	0.08
Native	129	141	LANVSTVLTSKYR	3	1450.81	4.60	0.83	2.69	0.09
Native	129	141	LANVSTVLTSKYR	3	1450.81	4.60	0.08	2.48	0.05
6M urea	34	46	LSFPTTKTYFPHF	3	1584.80	4.83	833.33	4.02	0.00
Native	34	46	LSFPTTKTYFPHF	3	1584.80	4.83	833.33	4.60	0.13
Native	34	46	LSFPTTKTYFPHF	3	1584.80	4.83	83.33	4.20	0.11
Native	34	46	LSFPTTKTYFPHF	3	1584.80	4.83	8.33	4.00	0.11
Native	34	46	LSFPTTKTYFPHF	3	1584.80	4.83	0.83	2.60	0.18
Native	34	46	LSFPTTKTYFPHF	3	1584.80	4.83	0.08	2.30	0.15
6M urea	34	80	LSFPTTKTYFPHFDLSHGSAQVKGHGAKVAAALTKAVEHLDDLPGAL	6	4942.58	3.86	833.33	16.42	0.00
Native	34	80	LSFPTTKTYFPHFDLSHGSAQVKGHGAKVAAALTKAVEHLDDLPGAL	6	4942.58	3.86	833.33	16.20	0.29
Native	34	80	LSFPTTKTYFPHFDLSHGSAQVKGHGAKVAAALTKAVEHLDDLPGAL	6	4942.58	3.86	83.33	13.70	0.44
Native	34	80	LSFPTTKTYFPHFDLSHGSAQVKGHGAKVAAALTKAVEHLDDLPGAL	6	4942.58	3.86	8.33	11.80	0.18
Native	34	80	LSFPTTKTYFPHFDLSHGSAQVKGHGAKVAAALTKAVEHLDDLPGAL	6	4942.58	3.86	0.83	9.40	0.10
Native	34	80	LSFPTTKTYFPHFDLSHGSAQVKGHGAKVAAALTKAVEHLDDLPGAL	6	4942.58	3.86	0.08	7.80	0.25
6M urea	136	141	LTSKYR	2	766.43	3.37	833.33	2.25	0.00
Native	136	141	LTSKYR	2	766.43	3.37	833.33	2.45	0.02
Native	136	141	LTSKYR	2	766.43	3.37	83.33	2.38	0.02
Native	136	141	LTSKYR	2	766.43	3.37	8.33	2.20	0.12
Native	136	141	LTSKYR	2	766.43	3.37	0.83	2.10	0.11
Native	136	141	LTSKYR	2	766.43	3.37	0.08	2.11	0.06
6M urea	84	106	SDLHAHKLRVDPVNFKLLSHSLL	5	2638.47	3.16	833.33	8.17	0.00
Native	84	106	SDLHAHKLRVDPVNFKLLSHSLL	5	2638.47	3.16	833.33	7.70	0.19
Native	84	106	SDLHAHKLRVDPVNFKLLSHSLL	5	2638.47	3.16	83.33	6.90	0.24
Native	84	106	SDLHAHKLRVDPVNFKLLSHSLL	5	2638.47	3.16	8.33	6.00	0.22
Native	84	106	SDLHAHKLRVDPVNFKLLSHSLL	5	2638.47	3.16	0.83	4.33	0.09
Native	84	106	SDLHAHKLRVDPVNFKLLSHSLL	5	2638.47	3.16	0.08	3.20	0.18
6M urea	81	106	SELSDLHAHKLRVDPVNFKLLSHSLL	5	2967.63	3.30	833.33	9.50	0.00
Native	81	106	SELSDLHAHKLRVDPVNFKLLSHSLL	5	2967.63	3.30	833.33	9.40	0.30

Continued on next page

Table S4 – *Continued from previous page*

Protein state	StaMT	End	Sequence	Charge	Peptide mass (Da)	MT (min)	HDX time (min)	Uptake (D)	Uptake SD (D)
Native	81	106	SELSDLHAHKLRVDPVNFKLLSHSLL	5	2967.63	3.30	83.33	8.68	0.08
Native	81	106	SELSDLHAHKLRVDPVNFKLLSHSLL	5	2967.63	3.30	8.33	7.84	0.03
Native	81	106	SELSDLHAHKLRVDPVNFKLLSHSLL	5	2967.63	3.30	0.83	5.90	0.12
Native	81	106	SELSDLHAHKLRVDPVNFKLLSHSLL	5	2967.63	3.30	0.08	4.87	0.04
6M urea	108	141	TLASHLPSDFTPAVHASLDKFLANVSTVLTSKYR	5	3685.95	4.37	833.33	14.15	0.00
Native	108	141	TLASHLPSDFTPAVHASLDKFLANVSTVLTSKYR	5	3685.95	4.37	833.33	11.00	0.10
Native	108	141	TLASHLPSDFTPAVHASLDKFLANVSTVLTSKYR	5	3685.95	4.37	83.33	8.10	0.37
Native	108	141	TLASHLPSDFTPAVHASLDKFLANVSTVLTSKYR	5	3685.95	4.37	8.33	6.60	0.24
Native	108	141	TLASHLPSDFTPAVHASLDKFLANVSTVLTSKYR	5	3685.95	4.37	0.83	4.80	0.11
Native	108	141	TLASHLPSDFTPAVHASLDKFLANVSTVLTSKYR	5	3685.95	4.37	0.08	4.05	0.06
6M urea	1	28	VLSAADKGNVKAAWGKVGGHAAEYGAEA	4	2726.38	3.97	833.33	10.88	0.00
Native	1	28	VLSAADKGNVKAAWGKVGGHAAEYGAEA	4	2726.38	3.97	833.33	11.57	0.09
Native	1	28	VLSAADKGNVKAAWGKVGGHAAEYGAEA	4	2726.38	3.97	83.33	11.20	0.40
Native	1	28	VLSAADKGNVKAAWGKVGGHAAEYGAEA	4	2726.38	3.97	8.33	10.20	0.12
Native	1	28	VLSAADKGNVKAAWGKVGGHAAEYGAEA	4	2726.38	3.97	0.83	7.30	0.12
Native	1	28	VLSAADKGNVKAAWGKVGGHAAEYGAEA	4	2726.38	3.97	0.08	5.90	0.35
6M urea	1	29	VLSAADKGNVKAAWGKVGGHAAEYGAEAL	4	2839.46	4.08	833.33	11.48	0.00
6M urea	1	29	VLSAADKGNVKAAWGKVGGHAAEYGAEAL	5	2839.46	4.08	833.33	10.89	0.00
Native	1	29	VLSAADKGNVKAAWGKVGGHAAEYGAEAL	4	2839.46	4.08	833.33	11.73	0.09
Native	1	29	VLSAADKGNVKAAWGKVGGHAAEYGAEAL	5	2839.46	4.08	833.33	11.30	0.21
Native	1	29	VLSAADKGNVKAAWGKVGGHAAEYGAEAL	4	2839.46	4.08	83.33	11.00	0.30
Native	1	29	VLSAADKGNVKAAWGKVGGHAAEYGAEAL	5	2839.46	4.08	83.33	10.90	0.47
Native	1	29	VLSAADKGNVKAAWGKVGGHAAEYGAEAL	4	2839.46	4.08	8.33	10.10	0.13
Native	1	29	VLSAADKGNVKAAWGKVGGHAAEYGAEAL	5	2839.46	4.08	8.33	9.79	0.02
Native	1	29	VLSAADKGNVKAAWGKVGGHAAEYGAEAL	4	2839.46	4.08	0.83	7.37	0.01
Native	1	29	VLSAADKGNVKAAWGKVGGHAAEYGAEAL	5	2839.46	4.08	0.83	7.20	0.15
Native	1	29	VLSAADKGNVKAAWGKVGGHAAEYGAEAL	4	2839.46	4.08	0.08	5.70	0.25
Native	1	29	VLSAADKGNVKAAWGKVGGHAAEYGAEAL	5	2839.46	4.08	0.08	5.40	0.33
6M urea	1	33	VLSAADKGNVKAAWGKVGGHAAEYGAEALERMF	5	3402.71	3.81	833.33	13.88	0.00
Native	1	33	VLSAADKGNVKAAWGKVGGHAAEYGAEALERMF	5	3402.71	3.81	833.33	12.10	0.42
Native	1	33	VLSAADKGNVKAAWGKVGGHAAEYGAEALERMF	5	3402.71	3.81	83.33	11.20	0.26
Native	1	33	VLSAADKGNVKAAWGKVGGHAAEYGAEALERMF	5	3402.71	3.81	8.33	10.00	0.23
Native	1	33	VLSAADKGNVKAAWGKVGGHAAEYGAEALERMF	5	3402.71	3.81	0.83	7.36	0.05
Native	1	33	VLSAADKGNVKAAWGKVGGHAAEYGAEALERMF	5	3402.71	3.81	0.08	5.60	0.19
6M urea	107	125	VTLASHLPSDFTPAVHASL	3	1962.02	5.73	833.33	5.89	0.00
Native	107	125	VTLASHLPSDFTPAVHASL	3	1962.02	5.73	833.33	4.50	0.22
Native	107	125	VTLASHLPSDFTPAVHASL	3	1962.02	5.73	83.33	3.80	0.16
Native	107	125	VTLASHLPSDFTPAVHASL	3	1962.02	5.73	8.33	3.50	0.26

Continued on next page

Table S4 – *Continued from previous page*

Protein state	StaMT	End	Sequence	Charge	Peptide mass (Da)	MT (min)	HDX time (min)	Uptake (D)	Uptake SD (D)
Native	107	125	VTLASHLPSDFTPAVHASL	3	1962.02	5.73	0.83	2.54	0.06
Native	107	125	VTLASHLPSDFTPAVHASL	3	1962.02	5.73	0.08	2.19	0.08
6M urea	107	128	VTLASHLPSDFTPAVHASLDKF	4	2352.21	4.84	833.33	6.95	0.00
Native	107	128	VTLASHLPSDFTPAVHASLDKF	4	2352.21	4.84	833.33	4.60	0.16
Native	107	128	VTLASHLPSDFTPAVHASLDKF	4	2352.21	4.84	83.33	3.70	0.18
Native	107	128	VTLASHLPSDFTPAVHASLDKF	4	2352.21	4.84	8.33	3.43	0.07
Native	107	128	VTLASHLPSDFTPAVHASLDKF	4	2352.21	4.84	0.83	2.40	0.12
Native	107	128	VTLASHLPSDFTPAVHASLDKF	4	2352.21	4.84	0.08	1.90	0.18
6M urea	107	136	VTLASHLPSDFTPAVHASLDKFLANVSTVL	4	3149.68	5.76	833.33	10.77	0.00
Native	107	136	VTLASHLPSDFTPAVHASLDKFLANVSTVL	4	3149.68	5.76	833.33	7.50	0.10
Native	107	136	VTLASHLPSDFTPAVHASLDKFLANVSTVL	4	3149.68	5.76	83.33	5.10	0.31
Native	107	136	VTLASHLPSDFTPAVHASLDKFLANVSTVL	4	3149.68	5.76	8.33	3.80	0.24
Native	107	136	VTLASHLPSDFTPAVHASLDKFLANVSTVL	4	3149.68	5.76	0.83	2.39	0.07
Native	107	136	VTLASHLPSDFTPAVHASLDKFLANVSTVL	4	3149.68	5.76	0.08	1.84	0.04
6M urea	107	141	VTLASHLPSDFTPAVHASLDKFLANVSTVLTSKYR	5	3785.01	4.39	833.33	14.45	0.00
Native	107	141	VTLASHLPSDFTPAVHASLDKFLANVSTVLTSKYR	5	3785.01	4.39	833.33	11.20	0.14
Native	107	141	VTLASHLPSDFTPAVHASLDKFLANVSTVLTSKYR	5	3785.01	4.39	83.33	8.10	0.33
Native	107	141	VTLASHLPSDFTPAVHASLDKFLANVSTVLTSKYR	5	3785.01	4.39	8.33	6.55	0.09
Native	107	141	VTLASHLPSDFTPAVHASLDKFLANVSTVLTSKYR	5	3785.01	4.39	0.83	4.90	0.11
Native	107	141	VTLASHLPSDFTPAVHASLDKFLANVSTVLTSKYR	5	3785.01	4.39	0.08	4.10	0.13

Table S5: HDX uptake for HBB_BOVIN with linear polyacrylamide coated fused-silica capillaries.

Protein state	StaMT	End	Sequence	Charge	Peptide mass (Da)	MT (min)	HDX time (min)	Uptake (D)	Uptake SD (D)
6M urea	134	145	AGVANALAHRYH	2	1278.66	3.63	833.33	4.38	0.00
Native	134	145	AGVANALAHRYH	2	1278.66	3.63	833.33	4.66	0.10
Native	134	145	AGVANALAHRYH	2	1278.66	3.63	83.33	4.10	0.16
Native	134	145	AGVANALAHRYH	2	1278.66	3.63	8.33	3.30	0.15
Native	134	145	AGVANALAHRYH	2	1278.66	3.63	0.83	1.80	0.27
Native	134	145	AGVANALAHRYH	2	1278.66	3.63	0.08	1.40	0.10
Native	114	132	ARNFGKEFTPVLQADFQKV	3	2194.15	4.38	833.33	8.40	0.00
Native	114	132	ARNFGKEFTPVLQADFQKV	3	2194.15	4.38	8.33	8.40	0.00
6M urea	114	145	ARNFGKEFTPVLQADFQKVVAGVANALAHRYH	6	3553.87	3.59	833.33	14.49	0.00
Native	114	145	ARNFGKEFTPVLQADFQKVVAGVANALAHRYH	6	3553.87	3.59	833.33	13.10	0.16
Native	114	145	ARNFGKEFTPVLQADFQKVVAGVANALAHRYH	6	3553.87	3.59	83.33	10.70	0.27
Native	114	145	ARNFGKEFTPVLQADFQKVVAGVANALAHRYH	6	3553.87	3.59	8.33	7.70	0.40
Native	114	145	ARNFGKEFTPVLQADFQKVVAGVANALAHRYH	6	3553.87	3.59	0.83	4.82	0.08
Native	114	145	ARNFGKEFTPVLQADFQKVVAGVANALAHRYH	6	3553.87	3.59	0.08	3.82	0.07
6M urea	52	84	AVMNNPKVKAHGKKVLDSFSNGMKHLDDLKGTF	6	3625.89	3.26	833.33	10.71	0.00
Native	52	84	AVMNNPKVKAHGKKVLDSFSNGMKHLDDLKGTF	6	3625.89	3.26	833.33	11.50	0.15
Native	52	84	AVMNNPKVKAHGKKVLDSFSNGMKHLDDLKGTF	6	3625.89	3.26	83.33	11.20	0.50
Native	52	84	AVMNNPKVKAHGKKVLDSFSNGMKHLDDLKGTF	6	3625.89	3.26	8.33	10.40	0.49
Native	52	84	AVMNNPKVKAHGKKVLDSFSNGMKHLDDLKGTF	6	3625.89	3.26	0.83	8.25	0.09
Native	52	84	AVMNNPKVKAHGKKVLDSFSNGMKHLDDLKGTF	6	3625.89	3.26	0.08	7.20	0.57
6M urea	51	84	DAVMNNPKVKAHGKKVLDSFSNGMKHLDDLKGTF	5	3740.91	3.32	833.33	10.82	0.00
Native	51	84	DAVMNNPKVKAHGKKVLDSFSNGMKHLDDLKGTF	5	3740.91	3.32	833.33	11.76	0.00
Native	51	84	DAVMNNPKVKAHGKKVLDSFSNGMKHLDDLKGTF	5	3740.91	3.32	83.33	11.60	0.53
Native	51	84	DAVMNNPKVKAHGKKVLDSFSNGMKHLDDLKGTF	5	3740.91	3.32	8.33	10.46	0.00
Native	51	84	DAVMNNPKVKAHGKKVLDSFSNGMKHLDDLKGTF	5	3740.91	3.32	0.83	8.23	0.10
Native	51	84	DAVMNNPKVKAHGKKVLDSFSNGMKHLDDLKGTF	5	3740.91	3.32	0.08	7.40	0.34
6M urea	128	145	DFQKVVAGVANALAHRYH	4	1995.04	3.63	833.33	7.59	0.00
Native	128	145	DFQKVVAGVANALAHRYH	4	1995.04	3.63	833.33	7.10	0.33
Native	128	145	DFQKVVAGVANALAHRYH	4	1995.04	3.63	83.33	6.30	0.33
Native	128	145	DFQKVVAGVANALAHRYH	4	1995.04	3.63	8.33	5.10	0.27
Native	128	145	DFQKVVAGVANALAHRYH	4	1995.04	3.63	0.83	3.11	0.03
Native	128	145	DFQKVVAGVANALAHRYH	4	1995.04	3.63	0.08	2.44	0.09
6M urea	41	84	FESFGDLSTADAVMNNPKVKAHGKKVLDSFSNGMKHLDDLKGTF	6	4795.37	3.65	833.33	15.24	0.00
Native	41	84	FESFGDLSTADAVMNNPKVKAHGKKVLDSFSNGMKHLDDLKGTF	6	4795.37	3.65	833.33	17.10	0.30
Native	41	84	FESFGDLSTADAVMNNPKVKAHGKKVLDSFSNGMKHLDDLKGTF	6	4795.37	3.65	83.33	16.40	0.39
Native	41	84	FESFGDLSTADAVMNNPKVKAHGKKVLDSFSNGMKHLDDLKGTF	6	4795.37	3.65	8.33	15.70	0.48

Continued on next page

Table S5 – *Continued from previous page*

Protein state	StaMT	End	Sequence	Charge	Peptide mass (Da)	MT (min)	HDX time (min)	Uptake (D)	Uptake SD (D)
Native	41	84	FESFGDLSTADAVMNNPKVKAHGKKVLDSFSNGMKHLDDLKGTF	6	4795.37	3.65	0.83	13.40	0.18
Native	41	84	FESFGDLSTADAVMNNPKVKAHGKKVLDSFSNGMKHLDDLKGTF	6	4795.37	3.65	0.08	11.40	0.40
6M urea	102	109	FKLLGNVL	2	902.56	5.28	833.33	2.63	0.00
Native	102	109	FKLLGNVL	2	902.56	5.28	833.33	2.52	0.04
Native	102	109	FKLLGNVL	2	902.56	5.28	83.33	2.08	0.04
Native	102	109	FKLLGNVL	2	902.56	5.28	8.33	1.32	0.02
Native	102	109	FKLLGNVL	2	902.56	5.28	0.83	0.51	0.04
Native	102	109	FKLLGNVL	2	902.56	5.28	0.08	0.25	0.03
6M urea	129	145	FQKVVAGVANALAHRYH	4	1880.02	3.34	833.33	7.33	0.00
Native	129	145	FQKVVAGVANALAHRYH	4	1880.02	3.34	833.33	6.80	0.26
Native	129	145	FQKVVAGVANALAHRYH	4	1880.02	3.34	83.33	6.00	0.50
Native	129	145	FQKVVAGVANALAHRYH	4	1880.02	3.34	8.33	4.90	0.16
Native	129	145	FQKVVAGVANALAHRYH	4	1880.02	3.34	0.83	3.10	0.14
Native	129	145	FQKVVAGVANALAHRYH	4	1880.02	3.34	0.08	2.50	0.20
6M urea	27	30	LGRL	1	457.30	3.95	833.33	0.97	0.00
Native	27	30	LGRL	1	457.30	3.95	833.33	0.77	0.01
Native	27	30	LGRL	1	457.30	3.95	83.33	0.61	0.03
Native	27	30	LGRL	1	457.30	3.95	8.33	0.60	0.03
Native	27	30	LGRL	1	457.30	3.95	0.83	0.52	0.00
Native	27	30	LGRL	1	457.30	3.95	0.08	0.46	0.01
6M urea	31	40	LVVYPWTQRF	2	1307.70	6.27	833.33	3.85	0.00
Native	31	40	LVVYPWTQRF	2	1307.70	6.27	833.33	2.83	0.04
Native	31	40	LVVYPWTQRF	2	1307.70	6.27	83.33	2.50	0.24
Native	31	40	LVVYPWTQRF	2	1307.70	6.27	8.33	2.30	0.12
Native	31	40	LVVYPWTQRF	2	1307.70	6.27	0.83	1.40	0.22
Native	31	40	LVVYPWTQRF	2	1307.70	6.27	0.08	1.10	0.10
6M urea	31	44	LVVYPWTQRFFESF	2	1817.91	7.53	833.33	5.05	0.00
Native	31	44	LVVYPWTQRFFESF	2	1817.91	7.53	833.33	4.43	0.07
Native	31	44	LVVYPWTQRFFESF	2	1817.91	7.53	83.33	4.00	0.22
Native	31	44	LVVYPWTQRFFESF	2	1817.91	7.53	8.33	3.60	0.14
Native	31	44	LVVYPWTQRFFESF	2	1817.91	7.53	0.83	2.60	0.15
Native	31	44	LVVYPWTQRFFESF	2	1817.91	7.53	0.08	2.25	0.09
6M urea	31	47	LVVYPWTQRFFESFGDL	3	2103.05	8.20	833.33	5.56	0.00
Native	31	47	LVVYPWTQRFFESFGDL	3	2103.05	8.20	833.33	5.07	0.04
Native	31	47	LVVYPWTQRFFESFGDL	3	2103.05	8.20	83.33	4.50	0.25
Native	31	47	LVVYPWTQRFFESFGDL	3	2103.05	8.20	8.33	3.90	0.35
Native	31	47	LVVYPWTQRFFESFGDL	3	2103.05	8.20	0.83	3.10	0.17
Native	31	47	LVVYPWTQRFFESFGDL	3	2103.05	8.20	0.08	2.77	0.04

Continued on next page

Table S5 – *Continued from previous page*

Protein state	StaMT	End	Sequence	Charge	Peptide mass (Da)	MT (min)	HDX time (min)	Uptake (D)	Uptake SD (D)
6M urea	1	13	MLTAEEKAAVTAF	2	1380.70	6.83	833.33	5.09	0.00
Native	1	13	MLTAEEKAAVTAF	2	1380.70	6.83	833.33	6.01	0.02
Native	1	13	MLTAEEKAAVTAF	2	1380.70	6.83	83.33	6.00	0.13
Native	1	13	MLTAEEKAAVTAF	2	1380.70	6.83	8.33	6.10	0.08
Native	1	13	MLTAEEKAAVTAF	2	1380.70	6.83	0.83	5.40	0.15
Native	1	13	MLTAEEKAAVTAF	2	1380.70	6.83	0.08	4.50	0.11
6M urea	1	30	MLTAEEKAAVTAFWGKVKVDEVGGEALGRL	5	3174.67	4.40	833.33	14.38	0.00
Native	1	30	MLTAEEKAAVTAFWGKVKVDEVGGEALGRL	5	3174.67	4.40	833.33	15.48	0.06
Native	1	30	MLTAEEKAAVTAFWGKVKVDEVGGEALGRL	5	3174.67	4.40	83.33	14.30	0.30
Native	1	30	MLTAEEKAAVTAFWGKVKVDEVGGEALGRL	5	3174.67	4.40	8.33	14.10	0.08
Native	1	30	MLTAEEKAAVTAFWGKVKVDEVGGEALGRL	5	3174.67	4.40	0.83	12.20	0.10
Native	1	30	MLTAEEKAAVTAFWGKVKVDEVGGEALGRL	5	3174.67	4.40	0.08	9.90	0.25
6M urea	138	145	NALAHRYH	2	980.49	3.65	833.33	4.00	0.00
Native	138	145	NALAHRYH	2	980.49	3.65	833.33	3.50	0.74
Native	138	145	NALAHRYH	2	980.49	3.65	83.33	3.60	0.40
Native	138	145	NALAHRYH	2	980.49	3.65	8.33	1.80	0.56
Native	138	145	NALAHRYH	2	980.49	3.65	0.83	1.50	0.32
Native	138	145	NALAHRYH	2	980.49	3.65	0.08	1.00	1.40
6M urea	116	135	NFGKEFTPVLQADFQKVVAG	3	2194.14	3.87	833.33	7.64	0.00
Native	116	135	NFGKEFTPVLQADFQKVVAG	3	2194.14	3.87	833.33	1.30	0.47
Native	116	135	NFGKEFTPVLQADFQKVVAG	3	2194.14	3.87	83.33	2.48	0.00
6M urea	72	95	NGMKHLDDLKGTFAALSELHCDKL	4	2655.31	3.93	833.33	10.08	0.00
Native	72	95	NGMKHLDDLKGTFAALSELHCDKL	4	2655.31	3.93	833.33	11.50	0.14
Native	72	95	NGMKHLDDLKGTFAALSELHCDKL	4	2655.31	3.93	83.33	10.80	0.25
Native	72	95	NGMKHLDDLKGTFAALSELHCDKL	4	2655.31	3.93	8.33	9.80	0.13
Native	72	95	NGMKHLDDLKGTFAALSELHCDKL	4	2655.31	3.93	0.83	7.10	0.27
Native	72	95	NGMKHLDDLKGTFAALSELHCDKL	4	2655.31	3.93	0.08	5.40	0.23
6M urea	35	40	PWTQRF	1	833.42	6.27	833.33	2.39	0.00
Native	35	40	PWTQRF	1	833.42	6.27	833.33	1.97	0.06
Native	35	40	PWTQRF	1	833.42	6.27	83.33	1.90	0.20
Native	35	40	PWTQRF	1	833.42	6.27	8.33	1.70	0.13
Native	35	40	PWTQRF	1	833.42	6.27	0.83	1.10	0.16
Native	35	40	PWTQRF	1	833.42	6.27	0.08	0.80	0.11
6M urea	126	145	QADFQKVVAGVANALAHRYH	4	2194.14	3.65	833.33	8.50	0.00
Native	126	145	QADFQKVVAGVANALAHRYH	4	2194.14	3.65	833.33	7.50	0.25
Native	126	145	QADFQKVVAGVANALAHRYH	4	2194.14	3.65	83.33	6.20	0.32
Native	126	145	QADFQKVVAGVANALAHRYH	4	2194.14	3.65	8.33	5.10	0.20
Native	126	145	QADFQKVVAGVANALAHRYH	4	2194.14	3.65	0.83	3.20	0.13

Continued on next page

Table S5 – *Continued from previous page*

Protein state	StaMT	End	Sequence	Charge	Peptide mass (Da)	MT (min)	HDX time (min)	Uptake (D)	Uptake SD (D)
Native	126	145	QADFQKVVAGVANALAHRYH	4	2194.14	3.65	0.08	2.50	0.11
6M urea	130	145	QKVVAGVANALAHRYH	4	1732.95	3.18	833.33	6.37	0.00
Native	130	145	QKVVAGVANALAHRYH	4	1732.95	3.18	833.33	6.60	0.38
Native	130	145	QKVVAGVANALAHRYH	4	1732.95	3.18	83.33	5.40	0.32
Native	130	145	QKVVAGVANALAHRYH	4	1732.95	3.18	8.33	4.26	0.09
Native	130	145	QKVVAGVANALAHRYH	4	1732.95	3.18	0.83	3.15	0.08
Native	130	145	QKVVAGVANALAHRYH	4	1732.95	3.18	0.08	1.90	0.32
6M urea	3	30	TAEEKAAVTAFWGKVKVDEVGGEALGRL	4	2930.55	4.23	833.33	13.27	0.00
Native	3	30	TAEEKAAVTAFWGKVKVDEVGGEALGRL	4	2930.55	4.23	833.33	14.37	0.08
Native	3	30	TAEEKAAVTAFWGKVKVDEVGGEALGRL	4	2930.55	4.23	83.33	13.30	0.34
Native	3	30	TAEEKAAVTAFWGKVKVDEVGGEALGRL	4	2930.55	4.23	8.33	13.10	0.19
Native	3	30	TAEEKAAVTAFWGKVKVDEVGGEALGRL	4	2930.55	4.23	0.83	11.40	0.12
Native	3	30	TAEEKAAVTAFWGKVKVDEVGGEALGRL	4	2930.55	4.23	0.08	9.30	0.32
6M urea	14	30	WGKVKVDEVGGEALGRL	3	1811.99	3.95	833.33	6.14	0.00
6M urea	14	30	WGKVKVDEVGGEALGRL	4	1811.99	3.96	833.33	6.13	0.00
Native	14	30	WGKVKVDEVGGEALGRL	3	1811.99	3.95	833.33	5.80	0.19
Native	14	30	WGKVKVDEVGGEALGRL	4	1811.99	3.96	833.33	5.80	0.05
Native	14	30	WGKVKVDEVGGEALGRL	3	1811.99	3.95	83.33	5.00	0.15
Native	14	30	WGKVKVDEVGGEALGRL	4	1811.99	3.96	83.33	5.00	0.14
Native	14	30	WGKVKVDEVGGEALGRL	3	1811.99	3.95	8.33	4.90	0.10
Native	14	30	WGKVKVDEVGGEALGRL	4	1811.99	3.96	8.33	4.90	0.10
Native	14	30	WGKVKVDEVGGEALGRL	3	1811.99	3.95	0.83	4.20	0.15
Native	14	30	WGKVKVDEVGGEALGRL	4	1811.99	3.96	0.83	4.13	0.10
Native	14	30	WGKVKVDEVGGEALGRL	3	1811.99	3.95	0.08	3.50	0.14
Native	14	30	WGKVKVDEVGGEALGRL	4	1811.99	3.96	0.08	3.50	0.15
6M urea	112	125	VLARNFGKEFTPVL	2	1589.89	5.15	833.33	5.46	0.00
Native	112	125	VLARNFGKEFTPVL	2	1589.89	5.15	833.33	3.74	0.06
Native	112	125	VLARNFGKEFTPVL	2	1589.89	5.15	83.33	3.70	0.42
Native	112	125	VLARNFGKEFTPVL	2	1589.89	5.15	8.33	3.20	0.34
Native	112	125	VLARNFGKEFTPVL	2	1589.89	5.15	0.83	2.10	0.28
Native	112	125	VLARNFGKEFTPVL	2	1589.89	5.15	0.08	1.60	0.14
6M urea	110	125	VVVLARNFGKEFTPVL	3	1788.03	5.15	833.33	5.96	0.00
Native	110	125	VVVLARNFGKEFTPVL	3	1788.03	5.15	833.33	4.30	0.14
Native	110	125	VVVLARNFGKEFTPVL	3	1788.03	5.15	83.33	4.00	0.25
Native	110	125	VVVLARNFGKEFTPVL	3	1788.03	5.15	8.33	3.60	0.20
Native	110	125	VVVLARNFGKEFTPVL	3	1788.03	5.15	0.83	2.30	0.15
Native	110	125	VVVLARNFGKEFTPVL	3	1788.03	5.15	0.08	1.70	0.19
6M urea	110	127	VVVLARNFGKEFTPVLQA	3	1987.13	5.54	833.33	6.95	0.00

Continued on next page

Table S5 – *Continued from previous page*

Protein state	StaMT	End	Sequence	Charge	Peptide mass (Da)	MT (min)	HDX time (min)	Uptake (D)	Uptake SD (D)
Native	110	127	VVVLARNFGKEFTPVLQA	3	1987.13	5.54	833.33	4.40	0.02
Native	110	127	VVVLARNFGKEFTPVLQA	3	1987.13	5.54	83.33	4.00	0.33
Native	110	127	VVVLARNFGKEFTPVLQA	3	1987.13	5.54	8.33	3.70	0.22
Native	110	127	VVVLARNFGKEFTPVLQA	3	1987.13	5.54	0.83	2.30	0.22
Native	110	127	VVVLARNFGKEFTPVLQA	3	1987.13	5.54	0.08	1.70	0.18
6M urea	110	128	VVVLARNFGKEFTPVLQAD	3	2102.15	5.85	833.33	7.05	0.00
Native	110	128	VVVLARNFGKEFTPVLQAD	3	2102.15	5.85	833.33	4.49	0.08
Native	110	128	VVVLARNFGKEFTPVLQAD	3	2102.15	5.85	83.33	4.10	0.26
Native	110	128	VVVLARNFGKEFTPVLQAD	3	2102.15	5.85	8.33	3.60	0.18
Native	110	128	VVVLARNFGKEFTPVLQAD	3	2102.15	5.85	0.83	2.20	0.23
Native	110	128	VVVLARNFGKEFTPVLQAD	3	2102.15	5.85	0.08	1.60	0.11
6M urea	110	129	VVVLARNFGKEFTPVLQADF	3	2249.22	6.04	833.33	7.47	0.00
Native	110	129	VVVLARNFGKEFTPVLQADF	3	2249.22	6.04	833.33	4.40	0.20
Native	110	129	VVVLARNFGKEFTPVLQADF	3	2249.22	6.04	83.33	4.00	0.36
Native	110	129	VVVLARNFGKEFTPVLQADF	3	2249.22	6.04	8.33	3.60	0.19
Native	110	129	VVVLARNFGKEFTPVLQADF	3	2249.22	6.04	0.83	2.30	0.16
Native	110	129	VVVLARNFGKEFTPVLQADF	3	2249.22	6.04	0.08	1.68	0.09
6M urea	110	145	VVVLARNFGKEFTPVLQADFQKVVAGVANALAHRYH	5	3964.16	4.38	833.33	17.05	0.00
Native	110	145	VVVLARNFGKEFTPVLQADFQKVVAGVANALAHRYH	5	3964.16	4.38	833.33	13.10	0.13
Native	110	145	VVVLARNFGKEFTPVLQADFQKVVAGVANALAHRYH	5	3964.16	4.38	83.33	10.40	0.44
Native	110	145	VVVLARNFGKEFTPVLQADFQKVVAGVANALAHRYH	5	3964.16	4.38	8.33	7.70	0.28
Native	110	145	VVVLARNFGKEFTPVLQADFQKVVAGVANALAHRYH	5	3964.16	4.38	0.83	4.69	0.09
Native	110	145	VVVLARNFGKEFTPVLQADFQKVVAGVANALAHRYH	5	3964.16	4.38	0.08	3.70	0.12

Table S6: Separation characteristics for bare fused-silica and linear polyacrylamide coated capillaries for different background electrolyte compositions, temperatures and separation voltages.*

Capillary coating	T (°C)	V (kV)	Background electrolyte	pH @ 20°C	Peptide	n	t (s)	n_c
Bare fused silica	−5	22	20%HAc, 10%ACN, 10%DMF	1.91	ATII	4	167(1.3)	47(6.6)
					ME		206(0.87)	
		20	20% DMF, 20% HAc	2.56	ATII	2	254(3.5)	49(5.3)
					ME		334(5.6)	
	0	20	25% ACN, 1% FA	2.23	ATII	3	109(8.1)	72(9.2)
					ME		143(11)	
					β130–145	19	127(5.4)	120(9.9)
					β31–47		213(11)	
Linear polyacrylamide	−5	20	10% HAc	2.17	ATII	3	335(1.6)	100(4.1)
					ME		689(4.6)	
	0	20	10% HAc	2.17	ATII	3	238(11)	107(16)
					ME		450(7.7)	
					α87–106	18	181(2.1)	175(2.9)
					β31–47		492(2.9)	

*Abbreviations: ACN, acetonitrile; ATII, angiotensin II; DMF, dimethylformamide; FA, formic acid; HAc, acetic acid; ME, Met-enkephalin; n, replicates; n_c, upper limit peak capacity; t, migration time. For t and n_c all values are given as mean (%RSD).

Table S7: Assessment of carryover based on inspection of the top 18 most abundant, the two most hydrophilic, and the two most hydrophobic Hb peptic peptides identified from PLGS.

Peptide	m/z	Intensity	
		Hb peptic digest	Subsequent separation
α136–141	384.24	1.35E+04	NaN
β102–109	452.29	1.51E+05	NaN
α99–106	455.79	2.01E+05	NaN
β129–145	471.26	8.52E+04	NaN
β128–145	499.76	1.30E+05	NaN
α84–106	528.90	9.42E+04	NaN
β126–145	549.79	1.96E+05	NaN
α107–128	589.31	4.01E+04	NaN
α81–106	594.73	6.60E+04	NaN
β52–84	605.65	5.79E+04	NaN
β1–30	636.14	4.97E+05	NaN
β31–40	654.86	2.25E+05	NaN
β49–85	668.02	8.52E+04	NaN
α47–80	676.37	9.59E+04	NaN
α1–29	711.11	2.21E+05	NaN
α107–141	758.40	5.42E+05	NaN
α107–136	788.67	1.26E+05	NaN
β110–145	794.24	1.41E+05	NaN
β41–84	800.56	6.51E+04	NaN
β35–40	834.45	5.52E+03	NaN

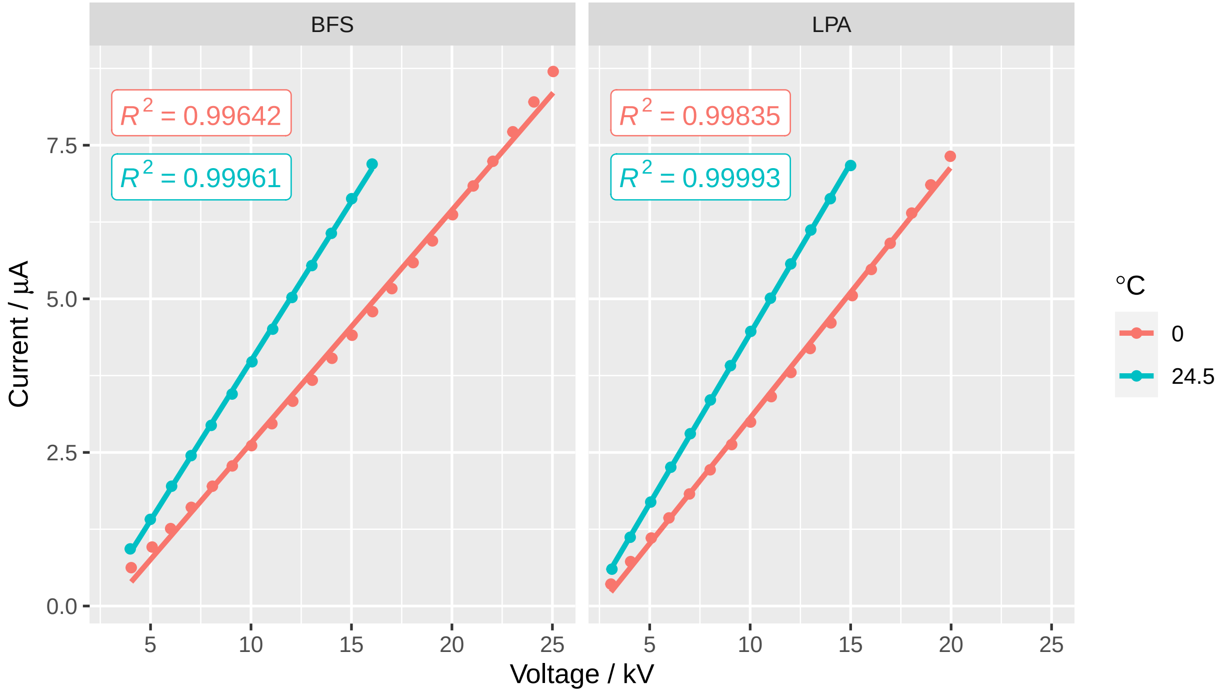

Figure S1: Ohm's plot for different capillary coatings and temperatures. In our experiments, at 0 °C, 20 kV separation voltage falls within the linear voltage–current response, indicating minimal Joule heating of the capillaries.

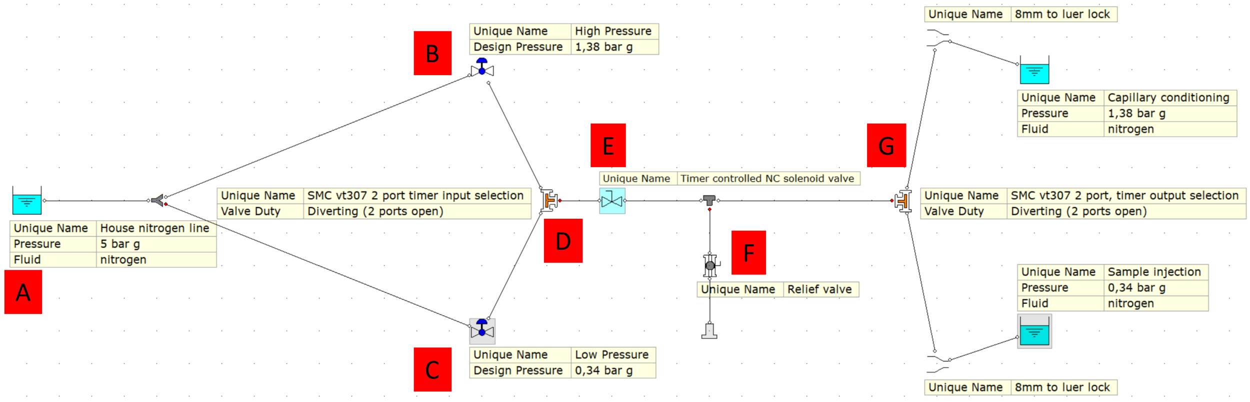

Figure S2: Schematic of the pneumatic system used for performing capillary conditioning and sample injection. **A**) A house line supplies N_2(g) at a pressure of 5 bar. This source is split into two pressure regulators. **B**) Pressure regulator dedicated for delivery of 1.38 bar "high pressure" N_2(g) to the acrylic pods used for solvent and sample delivery to the separation capillary. **C**) Pressure regulator dedicated for delivery of 0.34 bar "low pressure" N_2(g) to the acrylic pods used for solvent and sample delivery to the separation capillary. **D**) SMC VT307 diverting valve used to select whether high pressure or low pressure is feeding the system. **E**) A Crouzet Mcr1 timer connected to a Legrand 04453 pushbutton switch is used to open a valve that allows nitrogen to pressurize the rest of the downstream system. The timer is connected to a kill switch in case the wrong time is selected, the power to the timer can be interrupted, and then returned so the timer resets itself instead of the user having to wait the full length that was set on the timer. **F**) An RS PRO manual pneumatic control valve is used to quickly relieve pressure from the system after the timer shuts off. **G**) A second SMC VT307 diverting valve used for diverting the timer controlled solenoid outflow to one of two dedicated ports. The two diverting valves are controlled by the same rocker switch, such that selecting high pressure N_2(g) into the timer controlled solenoid valve, results in gas flow diversion to a dedicated high pressure port (shown as **1** in Figure S7A) where a pod can be connected for capillary conditioning.

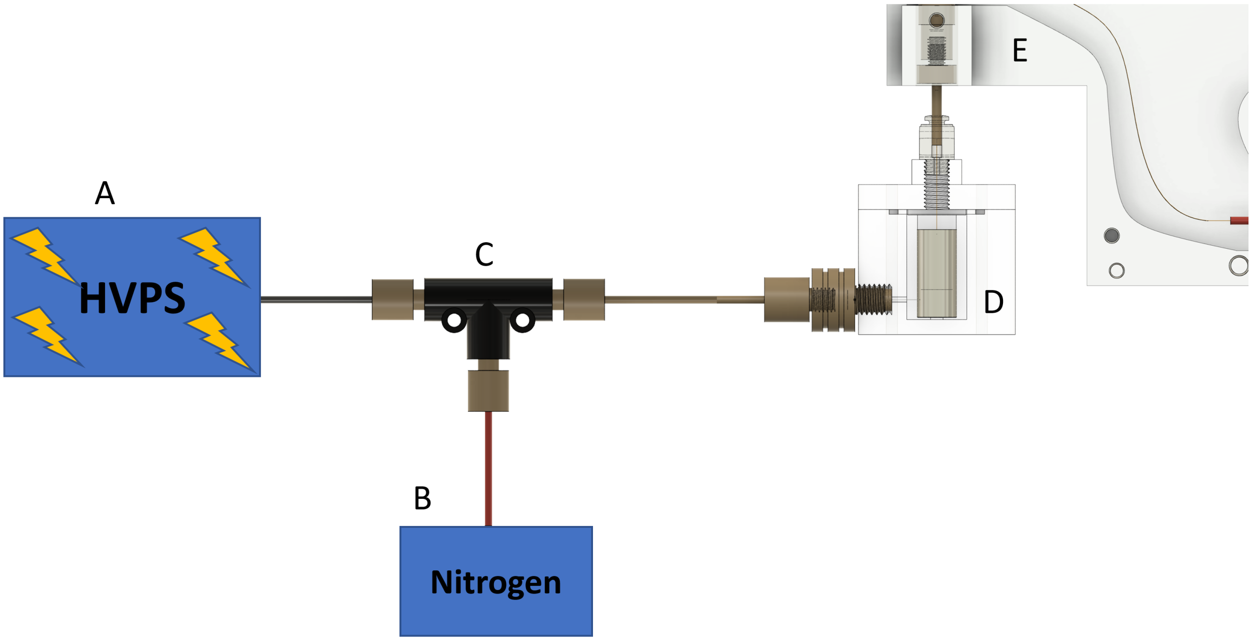

Figure S3: Diagram of major connections and fittings used for CE.**A**) High voltage power supply. **B**) N_2(g) source. **C**) P-727 PEEK tee assembly used to take HVPS load and N_2(g) inputs combined into a single output to provide separation voltage and N_2(g) for capillary conditioning to acrylic pod holding BGE vial (**D**). **E**) Inner face of cold side of CE device.

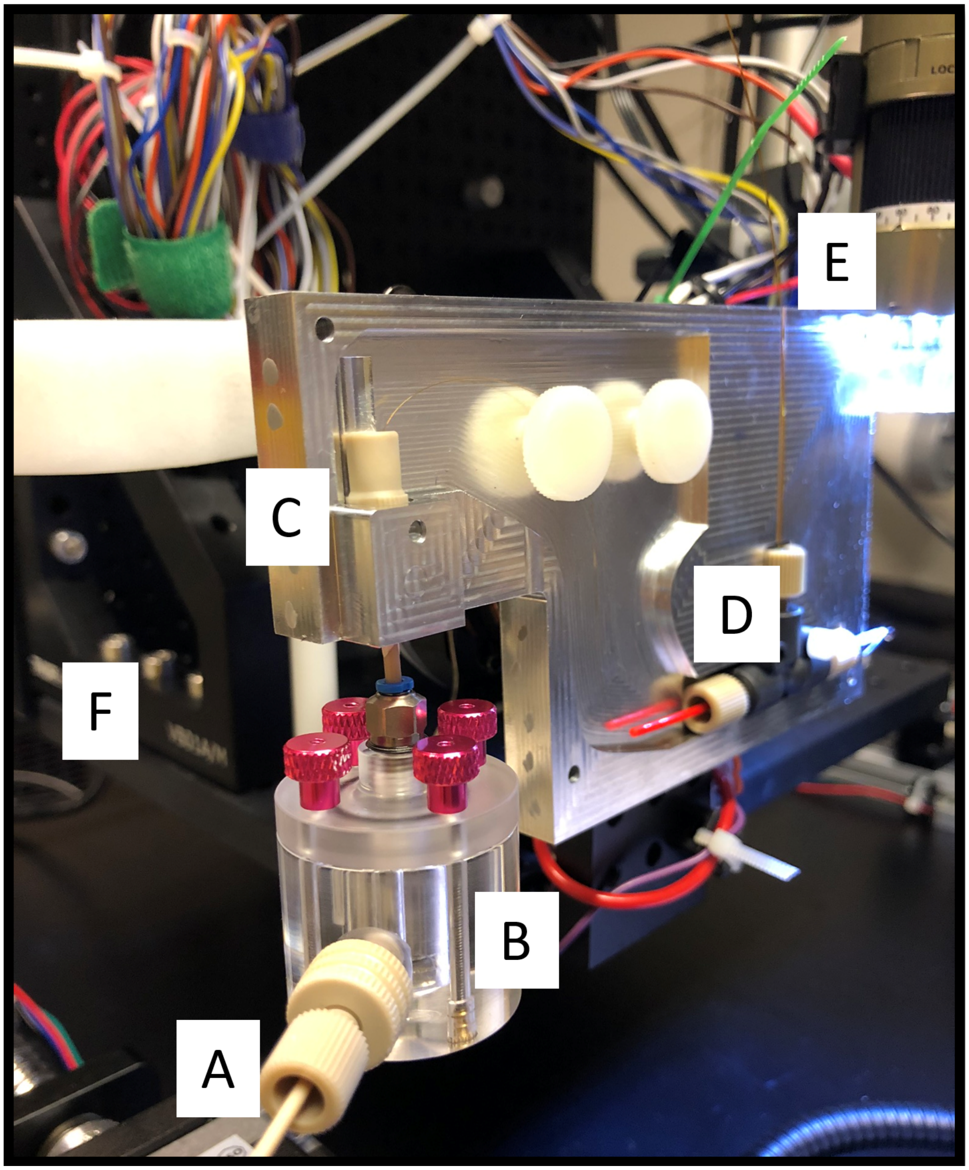

Figure S4: Image showing device with cover removed to highlight important components. **A**) 1/16" OD PEEK tubing delivering both high voltage load wire and nitrogen gas to gas tight BGE pod. PEEK tubing originates from part C Figure S3. **B**) Gas tight acrylic pod receiving N_2(g) (shown in greater detail in Figure S5), and high voltage load wire. A stainless steel vial is seated inside of the pod to hold BGE while allowing conduction of high voltage into the separation capillary. The separation capillary protrudes 3 mm below the bottom of the lid when the pod is attached to the device by 2 mm OD PEEK tubing connected to the P-720 MicroTight union with a F-120 ferrule. **C**) P-720 MicroTight union used to create a gas-tight seal at the capillary inlet, shown in more detail in Figure S8. **D**) Coaxial sheathflow nanoelectrospray emitter for ion generation at the capillary outlet. **E**) Long working distance digital microscope for assisting with positioning the emitter at the mass spectrometer inlet and monitoring electrospray stability. **F**) Part of the optical breadboard mounted on the XYZ stage used for positioning the device at the MS inlet.

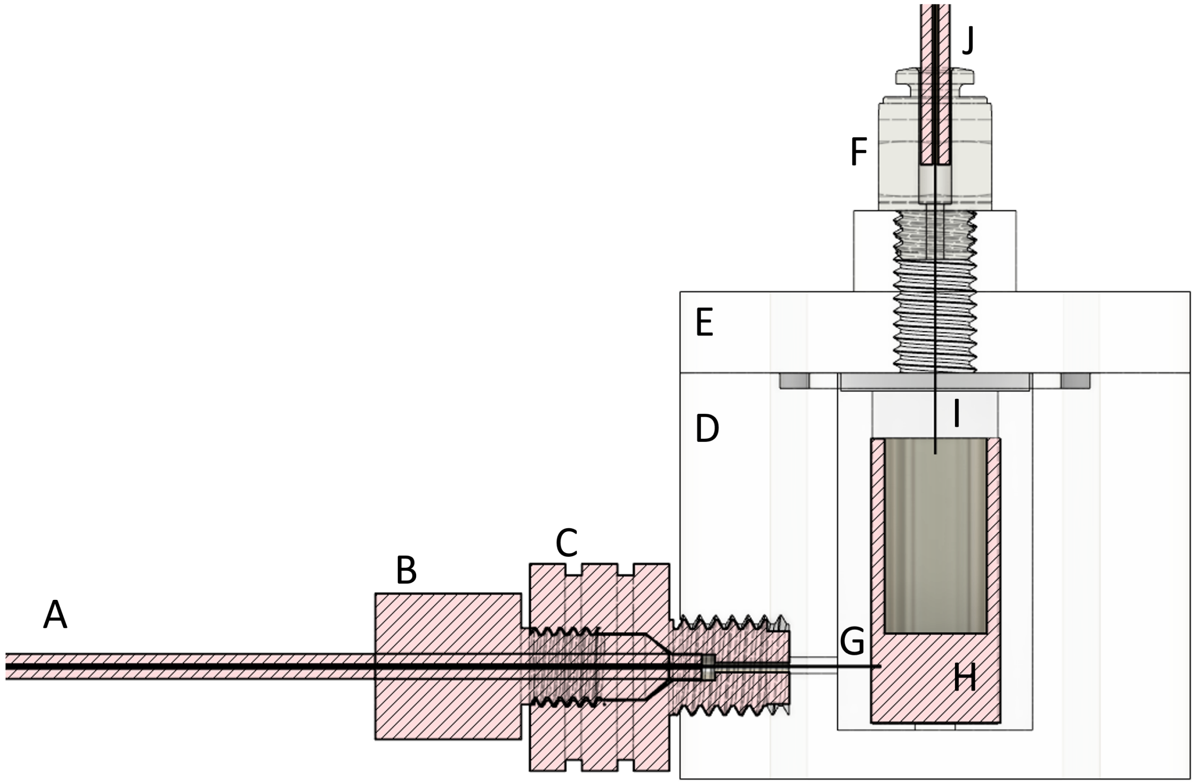

Figure S5: Close-up section representation of fittings used for gas- and electronic connections to BGE pod. With the exception of the Pt wire, the pod holding the sample vial is identical to the BGE pod. Electrical connections can easily be added to the sample pod if electrokinetic sample injection is desired. **A**) 1/16" OD PEEK tubing coming from tee in Figure S3 (part C). **B**) 1/16" PEEK nut, part F-120. **C**) PEEK adapter 10-32 female to M6 male, part P-920-01. **D**) Acrylic pod body with channel for an 15.5 x 1.3 mm NBR o-ring, a chamber to hold the BGE vial, and a 1 mm diameter through-hole to pass N_2(g) and the Pt wire coming through the P-920-01 adapter. **E**) Lid for acrylic pod with 2 mm push in to M5 gas fitting (**F**). **G**) 127 µm diameter platinum wire carrying HVPS load. **H**) Stainless-steel BGE vial. **I**) 40 µm ID, 105 µm OD fused silica capillary. **J**) 2mm PEEK tubing from capillary inlet assembly.

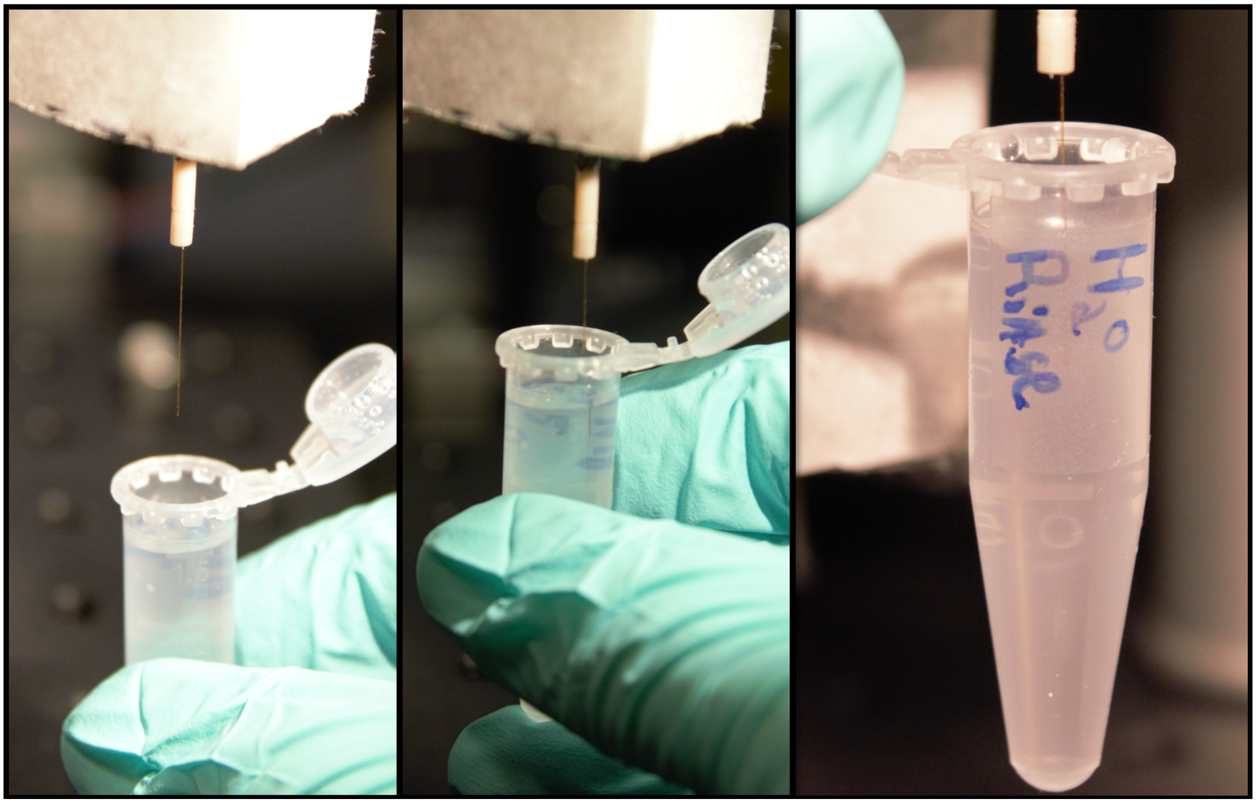

Figure S6: Capillary inlet rinsing to prevent carryover. Following sample injections and capillary conditioning steps, the capillary inlet is immersed into DI H_2O to rinse residual NaOH, proteins, or peptides from the outside of the separation capillary.

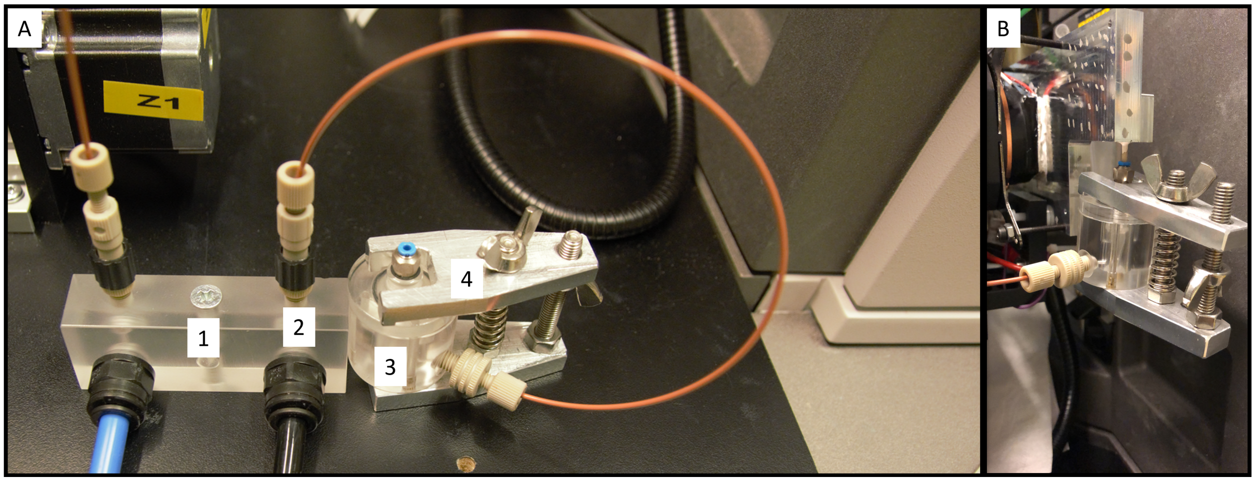

Figure S7: Sample injection pod connected to gas manifold. **A**. An image showing (**1**) the in house machined gas manifold using push in 8 mm to G 1/4 straight threaded adaptor to P-683 $\frac{1}{4}$-28 male to Luer-lock assembly which receives the output from the SMC VT307 diverting valve (Figure S2G) allowing for connection to the BGE and sample pods. **2**) Low pressure N_2(g) is delivered to the sample pod through the Luer-lock assembly by a P-659 10-32 female to female Luer fitting using an F-120 1/16" PEEK nut to secure the 1/16" OD PEEK tubing. **3**) Gas-tight sample pod receiving low pressure N_2(g) through 1/16" OD PEEK tubing secured to the pod using F-120 1/16" PEEK nut attached to a PEEK 10-32 female to male M6 adapter. **4**) Custom-made aluminum clamp that can quickly seal the sample pod to gas-tight after placing the labeled, quenched, digested (or any other) sample inside. **B**) Simulated sample injection shows gas tight sample pod attached to capillary inlet as occurs during sample injection. 1–2 mm of the capillary inlet will be immersed in the sample solution depending on how much sample is in the vial inside of the pod.

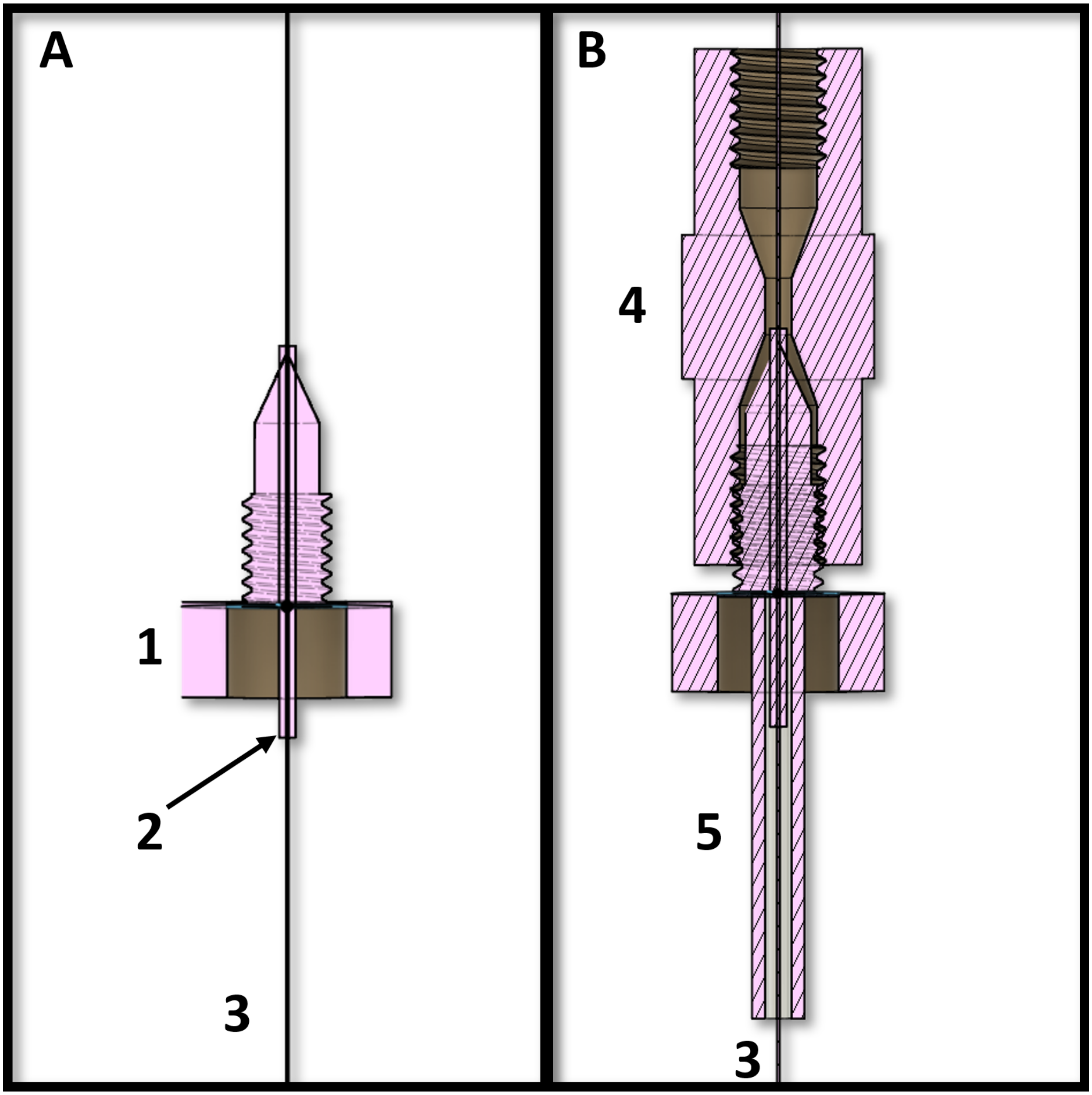

Figure S8: Section representation of separation capillary inlet assembly. Panel **A** shows the separation capillary (**3**), passed through F-180 MicroTight tubing sleeve (**2**) and into a micro fingertight PEEK 6-32 ferrule (**1**). Panel **B** shows this assembly screwed into a MicroTight PEEK union (**4**). A small length of 2 mm OD 0.5 mm ID PEEK tubing (**5**) is then pushed onto the MicroTight tubing sleeve and secured in the ferrule with epoxy. The capillary inlet (**3**) can now be inserted into one of the gas-tight pods by pushing the 2 mm peek tubing into the 2 mm push-in fitting (Figure S5F) and gas pressure introduced into the pods will produce the movement of fluid through the separation capillary towards the capillary outlet. This enables capillary conditioning, sample injection and use of the device as a variable temperature direct infusion electrospray source for volume-limited samples or instrument calibration.

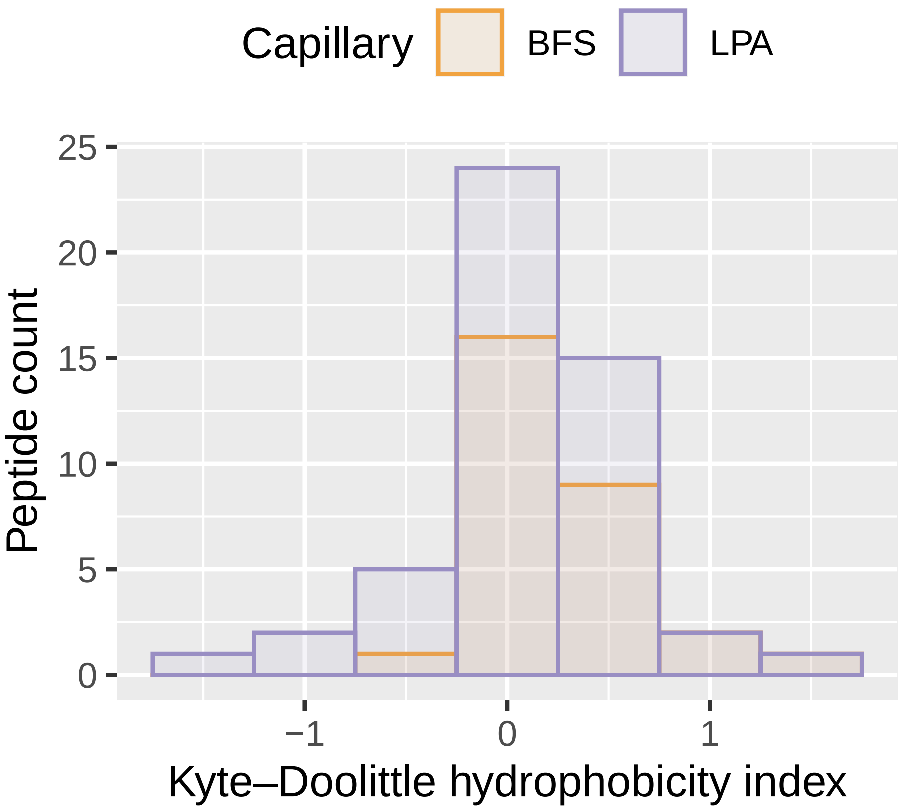

Figure S9: Hydrophobicity histogram for HDX-suitable peptic Hb peptides. Distribution of identified peptides used in the final HDX dataset based on their Kyte–Doolittle hydrophobicity values. Orange bars represent peptide signals of sufficient quality from separations on BFS capillaries to be used across the full HDX time course. Purple bars represent peptide signals of sufficient quality from separations on LPA capillaries to be used across the full HDX time course.

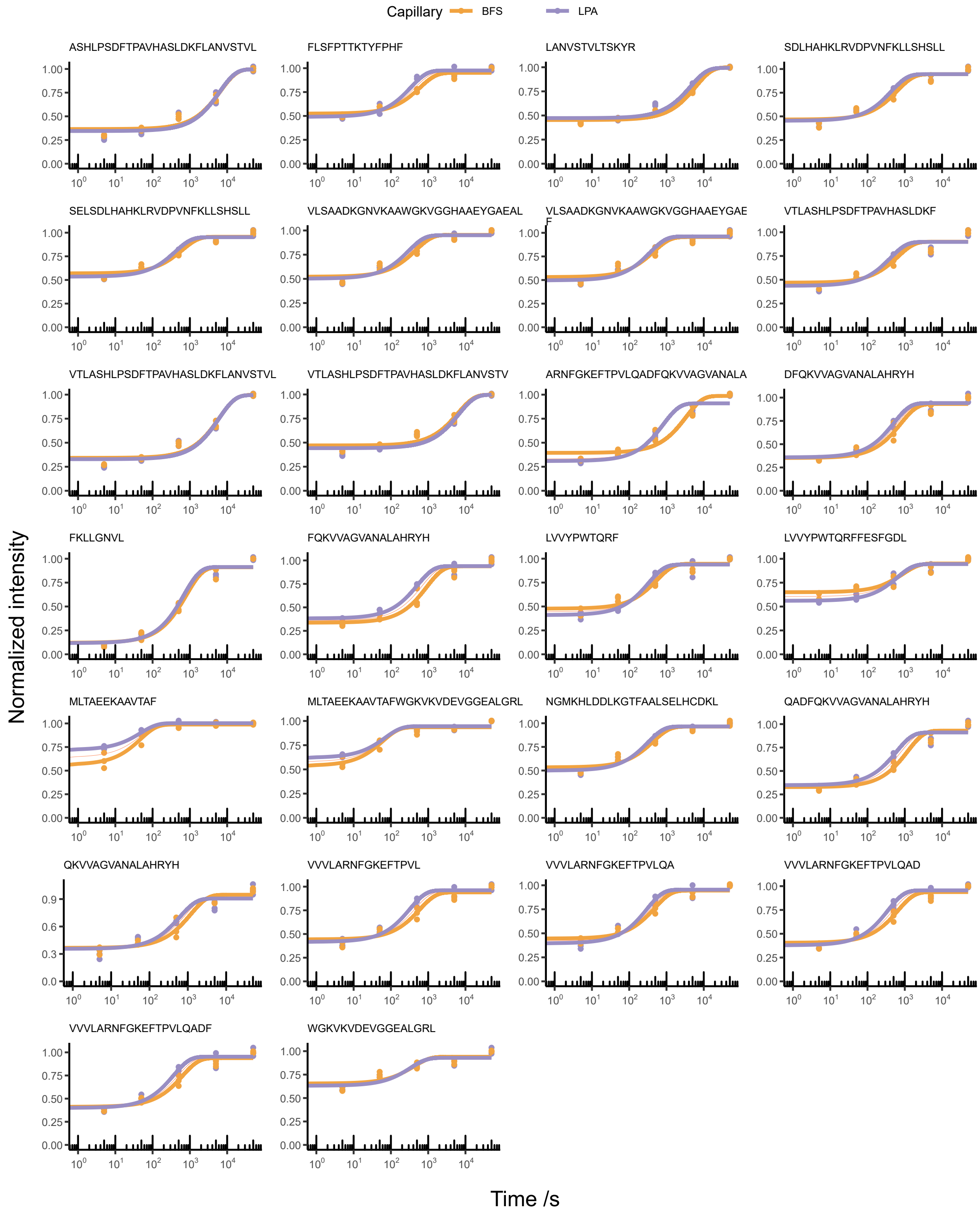

Figure S10: Deuterium uptake plots for bovine hemoglobin. Uptake of deuterium normalized against the maximum deuteron incorporation for each peptide per capillary type used for separation. No significant differences were observed across the uptake rates.

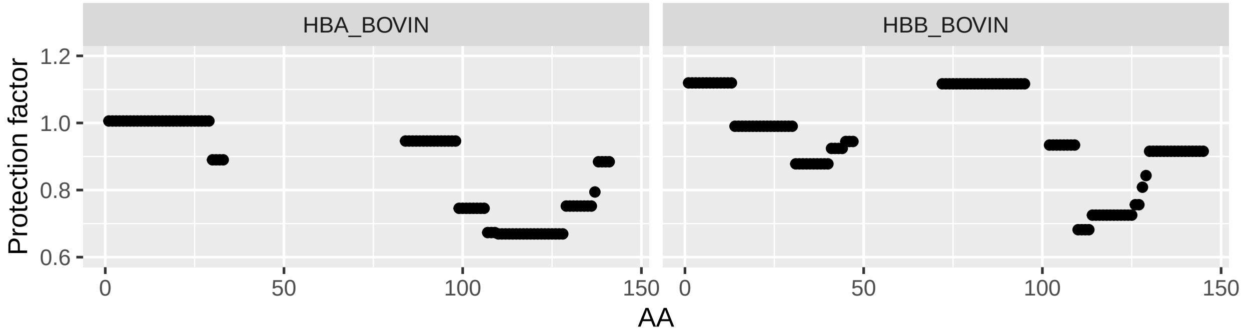

Figure S11: Relative protection plot. Relative protection determined as the average ratio of deuterium uptake under native conditions compared to denaturating conditions per amino-acid residue. These values were used for the false-coloring map in Figure 3.
